# A high-quality genome assembly of quinoa provides insights into the molecular basis of salt bladder-based salinity tolerance and the exceptional nutritional value

**DOI:** 10.1038/cr.2017.124

**Published:** 2017-10-10

**Authors:** Changsong Zou, Aojun Chen, Lihong Xiao, Heike M Muller, Peter Ache, Georg Haberer, Meiling Zhang, Wei Jia, Ping Deng, Ru Huang, Daniel Lang, Feng Li, Dongliang Zhan, Xiangyun Wu, Hui Zhang, Jennifer Bohm, Renyi Liu, Sergey Shabala, Rainer Hedrich, Jian-Kang Zhu, Heng Zhang

**Affiliations:** 1 Shanghai Center for Plant Stress Biology, CAS Center for Excellence in Molecular Plant Sciences, 3888 Chenhua Rd, Shanghai 201602, China;; 2 Julius-von-Sachs-Institut für Biowissenschaften, Biozentrum, University of Würzburg, D-97082 Würzburg, Germany;; 3 Plant Genome and Systems Biology, Helmholtz Center Munich, D-85764 Neuherberg, Germany;; 4 1gene Corporation, 88 Jucai Road, Hangzhou, Zhejiang 310050, China;; 5 Shanxi Jiaqi Quinoa Development Co. Ltd., Quinoa Industrial Park, Pinglu District, Shuozhou, Shanxi 038600, China;; 6 Key Laboratory of Plant Stress Research, Shandong Normal University, No. 88 Wenhua East Rd, Jinan, Shandong 250014, China;; 7 School of Land and Food, University of Tasmania, Hobart, TAS 7001, Australia;; 8 Department of Horticulture and Landscape Architecture, Purdue University, West Lafayette, IN 47907, USA

**Keywords:** quinoa, genome, halophyte, epidermal bladder cell

## Abstract

*Chenopodium quinoa* is a halophytic pseudocereal crop that is being cultivated in an ever-growing number of countries. Because quinoa is highly resistant to multiple abiotic stresses and its seed has a better nutritional value than any other major cereals, it is regarded as a future crop to ensure global food security. We generated a high-quality genome draft using an inbred line of the quinoa cultivar Real. The quinoa genome experienced one recent genome duplication about 4.3 million years ago, likely reflecting the genome fusion of two *Chenopodium* parents, in addition to the γ paleohexaploidization reported for most eudicots. The genome is highly repetitive (64.5% repeat content) and contains 54 438 protein-coding genes and 192 microRNA genes, with more than 99.3% having orthologous genes from glycophylic species. Stress tolerance in quinoa is associated with the expansion of genes involved in ion and nutrient transport, ABA homeostasis and signaling, and enhanced basal-level ABA responses. Epidermal salt bladder cells exhibit similar characteristics as trichomes, with a significantly higher expression of genes related to energy import and ABA biosynthesis compared with the leaf lamina. The quinoa genome sequence provides insights into its exceptional nutritional value and the evolution of halophytes, enabling the identification of genes involved in salinity tolerance, and providing the basis for molecular breeding in quinoa.

## Introduction

*Chenopodium quinoa* Willd. is an annual pseudocereal crop whose cultivation is expanding globally. During the past three decades, the number of countries growing this crop increased by 10-fold (from 8 in 1980 to 75 in 2014)^1^, mainly because quinoa is resistant to multiple abiotic stresses and the seed has exceptional nutritional qualities^2,3^. Quinoa was originally domesticated in the Andean region of South America as early as 7000 years ago^4^, and it is adapted to the harsh climatic conditions of the Andean area, which has a wide range of altitudes, temperature and annual precipitation. It is regarded as a facultative halophyte and shows a strong resistance to drought and low temperature as well^5,6^. The plant achieves the highest biomass when grown in the presence of 100 mM NaCl and shows only 20%∼50% biomass reduction even when treated with 500 mM NaCl (sea-water level)^7^. Compared to other grains, quinoa seeds lack gluten and have a better balanced ratio of proteins, lipids and carbohydrates, higher contents of essential amino acids and are rich in several minerals^2,8^. As a result, the National Aeronautics and Space Administration (NASA) of the United States considered quinoa as the optimal food source for astronauts in space^9^. The United Nations Food and Agricultural Organization (FAO) listed quinoa as an important crop to ensure global food security^10^ and declared 2013 as the International Year of Quinoa^1^.

Quinoa belongs to the flowering-plant family *Amaranthaceae*, which also contains sugar beet^11,12^, spinach and another pseudocereal, amaranth^13^. Quinoa is an allotetraploid (2n = 4× = 36) with an estimated genome size of approximately 1.5 Gbp^14,15^. Considered as a facultative autogamous plant, quinoa was reported to show heterogeneity and an outcrossing rate that ranges from 0.5% to 17%^16^. Despite having been domesticated for thousands of years, exceptional biodiversity is maintained in the current quinoa cultivars, through traditional seed exchanges of local Andean farmers^17^. The lack of breeding for specific environments, the high photoperiodic sensitivity and the relatively low yield are the major factors that limit quinoa cultivation in non-native areas^1^. Also, the genetic resources for this crop are limited^18,19^, which greatly hinders molecular marker-assisted breeding efforts. In order to develop high-density molecular markers and assist in the breeding process, a high-quality genome assembly of quinoa is needed.

Recently, quinoa has been studied as a model to understand salt tolerance in plants^3,20^. Epidermal bladder cells (EBCs), a cell structure homologous to trichomes, can be found in around 50% of all halophytes and are critical for their salt tolerance. In quinoa, the volume of EBCs is around 1 000 times of that of normal epidermal cells. EBCs are present when plants are not under salinity stress. The ion influxes of EBCs strongly increase in response to salt treatment and/or reactive oxygen species^3^. By engineering the trichome to salt bladder transition in glycophytic crop plants, salt-tolerant crops may be generated^3^. In order to use quinoa as a model halophytic plant and to study the molecular mechanism underlying salt bladder function and development, a high-quality genome draft is also important.

In this study, we generated a genome draft for *C. quinoa* using an inbred line of the quinoa Real, one of the most widely cultivated landraces in the world. We also generated high-depth transcriptome data from five representative types of quinoa tissues, and from EBCs with or without salt treatment. The expansion of gene families involved in stress response, ABA signaling and ion transport and a constitutive stress response at the transcript level were found to be correlated with the remarkable stress tolerance in quinoa. We also identified EBC-specific expression of ion transporters, H^+^-ATPases and sugar transporters supporting a model of polarized salt sequestration from leaf cells to EBCs^3^. The data presented here will facilitate studies to understand the molecular mechanisms of salt tolerance and help with the breeding of quinoa and the engineering of salt-tolerant crops.

## Results

### Phylogeny of *Chenopodium quinoa*

**Figure 1:**
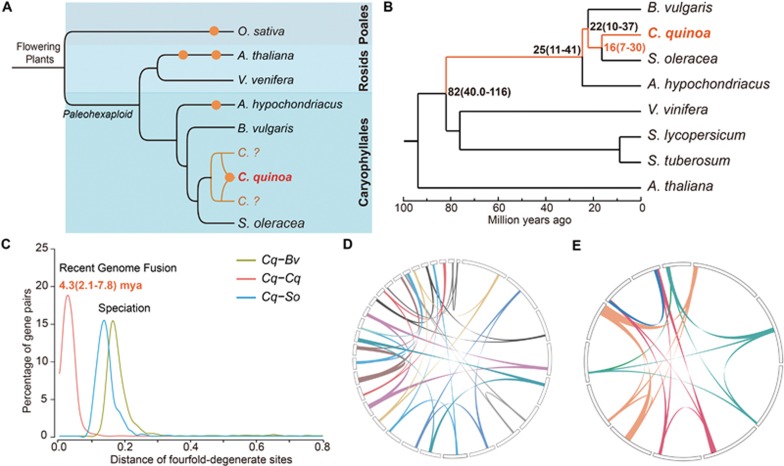
Evolution and synteny analyses of the *Chenopodium quinoa* genome. **(A)** Proposed evolution scenario of *C. quinoa*. The representative species from different orders are listed. Orders are indicated by different colors and order names are listed to the right. Orange points represent the event of whole-genome duplication or genome fusion. *C.*? represents the diploid parents that give rise to the allotetraploidic *C. quinoa*. **(B)** Phylogenetic analysis using selected plant species with published genome assemblies. It is estimated that *C. quinoa* and *Spinacia oleracea* were separated about 16 million years ago (MYA). **(C)** Four-fold-degenerate analyses suggest that the genome fusion event of *C. quinoa* (*Cq*) happened recently, after the divergence from spinach (*So*; *S. oleracea*) and sugar beet (*Bv*; *Beta vulgaris*). **(D)** Triplicated and **(E)** quadruplicated syntenic blocks in the *C. quinoa* genome.

Quinoa is a member of the plant family Amaranthaceae, which typically has a base chromosome number of 8–9. *C. quinoa* is believed to have been derived from the genome fusion of two related parent species from the same genus^15^. Amaranthaceae contains more than 2 000 known species, including some important economic plants, such as spinach (*Spinacia oleracea*), sugar beet (*Beta vulgaris*) and *Amaranthus hypochondriacus*^21^. Amaranthaceae is the largest family of the order Caryophyllales (Figure 1A)^21,22,23^, which represents more than 6% of angiosperm species diversity and is found in all terrestrial ecosystems^24^. Several independent lineages of C4 and CAM (crassulacean acid metabolism) plants, as well as a clade of carnivorous plants, belong to this order^21^. Despite an extraordinary diversity within Caryophyllales, genome sequences are available only for a limited number of species^12,13^ (Figure 1A). Caryophyllales and Rosids belong to the clade of Pentapetalae, which shares a paleohexaploid ancestor (Figure 1A) and is estimated to have a crown age of 104-113 MYA^23^.

### Genome sequencing and assembly

An inbred line of *C. quinoa* f. *real* that showed a low heterozygosity ratio in a pilot survey was used for genome sequencing (Supplementary information, Figure S1). Using a K-mer distribution analysis, we estimated that quinoa has a genome size of apporoximately 1.45 Gbp (Supplementary information, Figure S1), which is consistent with previous estimates based on C-values^14^.

We took a hybrid approach for genome assembly, combining 284 Gb of paired-end reads from HiSeq2500 (Illumina) and 50 Gb of single-molecule long reads from RS II (PacBio) (Supplementary information, Table S1). After filtering out low-quality bases, a total of 153 Gb of data from two small-size PCR-free libraries (∼80× coverage), two medium-size mate pair libraries (5 and 8 kb; ∼30× coverage) and one 20-kb PacBio library (∼34× coverage), were used for genome assembly (Supplementary information, Table S1). The final v1.0 assembly (Cq_real_v1.0) has a total length of 1 337 Mbp with a scaffold N50 equaling 1.16 Mbp (Table 1). The assembly covers 90.2% of the nuclear genome based on our estimation. Importantly, 90% of the assembly falls into 1 087 scaffolds that are at least 423 Kbp in length (Supplementary information, Table S1), with the largest scaffold being 5.4 Mbp. By utilizing the high coverage of single-molecule long reads on organelle genomes, we assembled the chloroplast genome into one single contig with a length of 152 282 bp (Supplementary information, Figure S2). However, a similar attempt failed to generate a complete genome for the mitochondrion.

In order to assess the quality of Cq_real_v1.0, we assembled 234 311 transcripts (> 200 bp) *de novo* from 60-Gb mRNA-seq data of five different quinoa tissues; 98.9% of these could be aligned to the assembly with more than 95% identity, demonstrating that the majority of gene space was covered (Supplementary information, Table S3). More than 98% of the Illumina reads from the two PCR-free libraries can be aligned back to the final assembly. After the alignment, 34 197 SNPs and 41 269 InDels were identified. 90% of the InDels had a size of less than 10 bp. That allowed us to estimate the consensus error rate as 0.0056%. Comparison to a published quinoa-genetic map containing 511 SNP (single-nucleotide polymorphism) markers, allowed 485 of our scaffolds to be anchored to 497 SNP markers by allowing one mismatch (Supplementary information, Figure S3)^25^. We also generated a quinoa fosmid library and pooled 10 random fosmids for high-depth single-molecule sequencing. After *de novo* assembly, we obtained 10 contigs corresponding to the 10 fosmids, all of which can be globally aligned to a specific scaffold with greater than 98% identity (Supplementary information, Table S4). These metrics indicate that our assembly is of high quality and has a low error rate.

### Genome annotation

We annotated the repeats in the genome by combining *in silico* prediction and homology searches. As much as 64.5% of the *C. quinoa* genome comprised various types of repeat sequences (Table 1, Supplementary information, Table S5), 85.6% of which are transposable elements (TEs). Retrotransposons comprise the majority of the TEs. LTR-type (long terminal repeat) transposons *Gypsy* and *Copia*, and the DNA transposon CMC are the three most abundant transposon families, making up 33.58%, 11.69% and 3.64% of the total genome content, respectively. Simple sequence repeats (SSRs) are usually polymorphic and provide a rich source of molecular markers for breeding and genetic studies. A total of 392 764 SSRs (Supplementary information, Table S6), ranging from mononucleotide to hexanucleotide motifs and representing 38 distinctive motif families, were identified and annotated in the *C. quinoa* genome (Supplementary information, Table S7).

We performed gene prediction by combining the results obtained from *ab initio* prediction, homology searches and transcriptome assembly, after masking the repetitive sequences (Supplementary information, Table S8). A total of 54 438 protein-coding genes were identified in the *C. quinoa* genome, that had an average length of 3 548 bp and contained an average of 4.8 exons per gene (Table 1). We also identified 192 micro RNAs (miRNAs), 1 310 ribosomal RNAs (rRNAs), 2 934 transfer RNAs (tRNAs) and 5 922 small nuclear RNAs (snRNAs) (Table 1, Supplementary information, Table S9). We assigned the functions to 95.6% of the protein-coding genes based on functional annotations from five different public databases (Supplementary information, Table S10). Remarkably, 75.4% of quinoa proteins showed a homology to the InterPro database, and 85.0% were identified in the gene ontology (GO) database (Supplementary information, Table S10).

Our predicted gene set covers 93.3% of the 1 440 single-copy orthologous genes in BUSCO v2 (Supplementary information, Table S11)^26^. More than 50% of the predicted gene models have a probabilistic confidence score of 1.0, indicating that evidence from *de novo* prediction, homology-based gene prediction and mRNA-seq data are mostly consistent (Supplementary information, Figure S4A; see Materials and Methods section for details). Indeed > 76% of the predicted genes with a confidence score greater than 0.5 (corresponding to ∼95% of all genes) contain at least one conserved protein domain (Supplementary information, Figure S4B). Over 96% of the annotated genes had supporting mRNA-sequencing reads (Supplementary information, Figure S4C). Using 56 quinoa mRNA sequences available in the NCBI nucleotide database, we estimated the specificity and sensitivity at the exon level to be 69.74% and 43.09%, respectively. This estimation of exon specificity is consistent with the estimation of exon sensitivity (71.92%) using another set of high-expression transcripts assembled from mRNA-seq data (Supplementary information, Table S12). These results indicate a high accuracy of predicted gene models in Cq_real_v1.0.

We compared Cq_real_v1.0 and two recently published quinoa genome drafts. While all three assemblies have consistent estimates of the GC ratio and the repeat content, the two assemblies utilizing single-molecule long reads clearly have a better coverage and contiguity than the one using only short reads (Supplementary information, Table S13)^27,28^. ASM168347v1 has a similar number of scaffolds and covers a similar number of bases as Cq_real_v1.0 (∼1 325 Mbp), but has a higher scaffold of N50 length and a smaller number of N50 scaffolds (Supplementary information, Table S13)^28^. On the other hand, Cq_real_v1.0 includes 9 693 more gene models while covering approximately 98.5% (44 124 of 44 776) of the predicted genes in ASM168347v1 (Supplementary information, Table S13). Most of the 10 554 Cq_real_v1.0-specific gene models have supporting mRNA-seq data and 78.3% of them are expressed (RPKM > 2) in at least 1 of the 11 types of quinoa samples examined (Supplementary information, Figure S5).

Comparative analyses of *C. quinoa* with *Arabidopsis thaliana*^29^, and three other species from the *Amaranthaceae* family, *A. hypochondriacus*^13^, *S. oleracea* and *B. vulgaris*^12^ indicated that the five plant species possess similar numbers of orthologous groups, with a core set of 10 538 in common (Supplementary information, Figure S6). Out of the 14 707 *C. quinoa* gene families, 14 492 were identified at least once in the other four plant genomes. Among the 315 quinoa-specific gene orthologous groups, 36.5% (825 genes) were predicted with an unknown function, while genes involved in auxin efflux transporter activity and the response to molecule of fungal origin were among the most significantly overrepresented (Supplementary information, Table S14).

### Genome evolution of quinoa

Cytological and molecular evidence suggests that the quinoa genome is the result of genome fusion between two different parent species of *Chenopodium*, each contributing to about half of the genome size^15^. Using the available genome sequences from the related species, we are able to identify the key time points in quinoa genome evolution. By examining single-copy gene families from eight sequenced plant genomes, we found that both quinoa and spinach (*S. oleracea*), belonging to a subfamily *Chenopodiaceae*, diverged some 16 million years ago, and shared a common ancestor with *A. hypochondriacus*, diverging about 25 million years ago (Figure 1B). Caryophylales and Rosids diverged about 82 million years ago. Consistent with previous studies, our phylogenetic analyses strongly suggest that Caryophyllales represents the most basal eudicot clade (Figure 1B)^12^.

We analyzed the fourth degenerate sites (4DTv) to identify the critical time points in quinoa genome evolution. We first identified 1 224 colinear blocks in the quinoa genome, which corresponds to approximately 31% (16 864/54 438) of the total gene set. Age distribution was calculated using 9 890 paralogous gene pairs of similar age after excluding local duplications. We observed a sharp peak centered on 4DTv around 0.028 indicating a recent whole-genome duplication (WGD) event (Figure 1C) that likely reflects the genome fusion between two parent *Chenopodium* species. The time of fusion is about 4.3 million years ago, which is much later than the divergence between quinoa and spinach or between quinoa and sugar beet. Within the quinoa genome, we also identified colinear blocks that corresponded to three or more different genomic regions (Figure 1D and 1E), which likely reflects the γ paleohexaploidization event observed in many eudicots. Considering that self-colinear blocks may retain a limited ancient colinearity, we used 16 481 paralogous gene pairs from the quinoa genome and calculated the *K*_s_ value (substitution per synonymous site) distribution. We observed two peaks of *K*_s_ at 0–0.2 and 1.2–1.8, respectively (Supplementary information, Figure S7). The first peak likely indicates the recent genome fusion event, while the second peak corresponds to the γ paleohexaploidization event^30,31^. Thus, besides the genome fusion event that happened around 4.3 million years ago, no other lineage-specific WGD events were involved in quinoa genome evolution (Figure 1A). This is similar to what was observed for the *B. vulgaris* genome^12^.

### Quinoa as a lysine-rich pseudocereal

**Figure 2:**
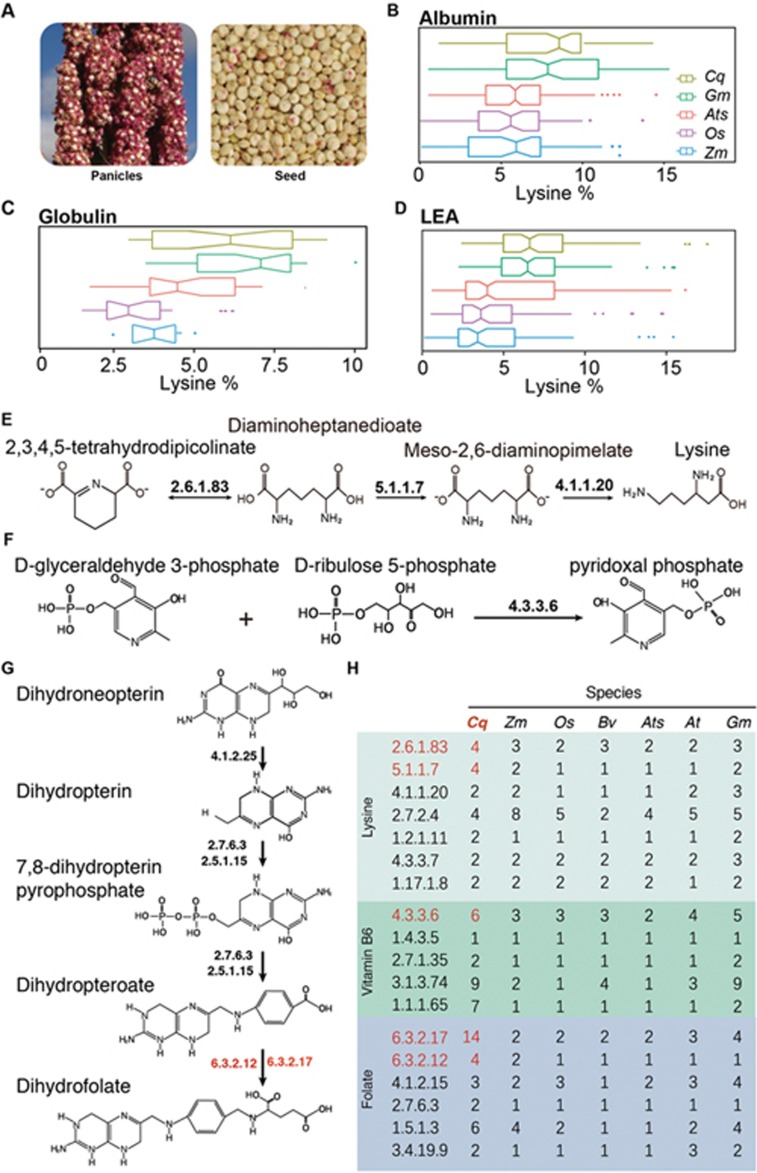
Gene copy number expansion associated with nutrient synthesis in the quinoa genome. **(A)** Photos of mature panicles (left) and dry seeds (right) of quinoa. **(B**-**D)** The sequences of quinoa seed storage proteins contain a significantly higher proportion of lysine residues compared to other cereals. The boxplots show the distribution of lysine percentage (% Lys – number of lysine residues divided by the total length of proteins) in the sequences of indicated protein families: **(B)** albumin, **(C)** globulin and **(D)** LEA (late embryogenesis abundant). **(E**-**G)** Diagrams showing the key steps in the biosynthesis of lysine **(E)**, vitamin B6 **(F)** and folate **(G)** in plants. The arrow line indicates the direction of biochemical reactions, and enzymes catalyzing the corresponding reactions are listed as Enzyme Commission (EC) numbers next to the arrow. **(H)** Gene copy number of enzymes involved in the biosynthesis of lysine, vitamin B6 and folate in selected plant species. The enzymes that show an expansion in the quinoa genome are colored red. Species are indicated by two-letter names: *At*, *Arabidopsis thaliana*, *Ats*, *Aegilops tauschii*, *Bv*, sugar beet (*Beta vulgaris*), *Cq*, quinoa (*Chenopodium quinoa*), *Gm*, soybean (*Glycine max*), *Os*, rice (*Oryza sativa*) and *Zm*, maize (*Zea mays*).

As a pseudocereal, quinoa is valued for its high protein content and richness in essential amino acids (Figure 2A). For example, lysine content is usually limited in cereal grains but plentiful in legumes^32^. We analyzed three families of candidate seed storage proteins, including albumin, globulin and late embryo abundant (LEA) proteins. We found that the lysine content of quinoa in all three protein families was significantly higher in comparison to the other cereals, such as wheat, rice or maize, to a level that was equivalent to soybeans (Figure 2B-2D). The consistently higher contents in all seed storage proteins were not observed for threonine or methionine, which share the same precursor as lysine for biosynthesis. We also observed a significantly higher usage of phenylalanine and isoleucine in all the seed storage proteins compared to other cereals (Supplementary information, Figures S8, S9, S10 and Table S16). Thus, the high contents of essential amino acids in quinoa are not only reflected at the level of free amino acids, but also at the level of amino acid usage in seed protein sequences.

We examined the key enzymes involved in lysine biosynthesis based on the KEGG annotation. There are seven enzymes involved in converting aspartate into lysine (Figure 2E and 2H). The copy numbers of two downstream enzymes, diaminopimelate aminotransferase (DAPAT; EC 2.6.1.83) and diaminopimelate epimerase (DAPE; EC 5.1.1.7), are higher than those in several cereals or sugar beet (Figure 2H).

Quinoa has also been reported to contain high levels of vitamin B and vitamin E^33^. Thus, we examined the key enzymes involved in vitamin biosynthesis (Figure 2F and 2G). One of the enzymes involved in vitamin B6 biosynthesis (pyridoxal 5′-phosphate synthase; EC 4.3.3.6) and two enzymes that generate dihydrofolate (dihydrofolate synthase, EC 6.3.2.12; tetrahydrofolate synthase, EC 6.3.2.17) have higher gene copy numbers in quinoa than in other plant species (Figure 2H).

### Enhanced ABA signaling and biosynthesis

**Figure 3:**
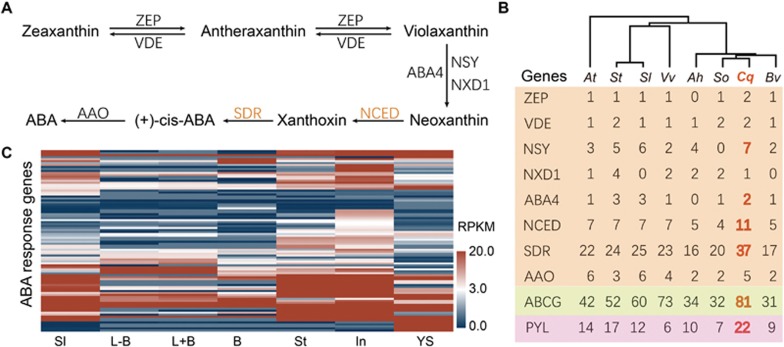
Genes involved in ABA homeostasis and perception are expanded in quinoa. **(A)** Diagram of the ABA biosynthetic pathway. The arrows indicate the direction of biochemical reactions. Enzymes that catalyze the reaction are indicated next to the arrow: ZEP, zeaxanthin epoxidase; VDE, violaxanthin de-epoxidase; ABA4, abscisic acid-deficient 4; NSY, neoxanthin synthase; NXD1, neoxanthin-deficient 1; NCED, 9-*cis*-epoxycarotenoid dioxygenases; SDR, short-chain dehydrogenases/reductases; AAO, aldehyde oxidase. **(B)** Copy number of genes involved in ABA biosynthesis, transport and perception in quinoa and seven other plant species. Red numbers indicate gene copy numbers that are higher in quinoa. The phylogenetic relationship of these species is indicated at the top of the graph, inferred from single-copy orthologous gene sets. Genes involved in ABA biosynthesis, transport and perception are shaded in orange, yellow and pink color, respectively. **(C)** Heatmap showing the transcript level of ABA-responsive genes across different quinoa tissues. Functional characterization is based on the GO (gene ontology) annotation. In total, 108 genes in quinoa genome were assigned as a “response to ABA”, and 92 of those (85%) are expressed in at least one of the seven types of tissues. Tissues are indicated in short names: Sl, seedlings; L − B, leaf without bladders; L + B, leaf with bladders; B, bladder cells; St, stem; In, inflorescence; YS, young seeds. Species are indicated by two-letter names: *Ah*, *Amaranthus hypochondriacus*; *At*, *Arabidopsis thaliana*; *Bv*, sugar beet (*Beta vulgaris*); *Cq*, quinoa (*Chenopodium quinoa*); *Sl*, tomato (*Solanum lycopersicum*); *So*, spinach (*Spinacia oleracea L.*); *St*, potato (*Solanum tuberosum*); *Vv*, grape (*Vitis vinifera*).

In order to understand quinoa's remarkable tolerance to salinity, drought and low temperature, we examined ABA-related genes in quinoa. Through BLAST-conserved domain search and manual curation, we identified the orthologs involved in ABA biosynthesis, transport and perception. We found that the key factors in each category were expanded in the quinoa genome. ABA biosynthesis in plants starts from the precursor isopentenyl diphosphate (IPP) in the plastid and proceeds through the carotenoid pathway (Figure 3A). The step catalyzed by NCEDs (9-*cis*-epoxycarotenoid dioxygenases), which cleave neoxanthin and produce the C_15_ intermediate xanthoxin, is considered to be the rate-limiting step for ABA biosynthesis. Quinoa contains 11 NCEDs, while other Caryophyllales plant species contain 4-5 (Figure 3B; Supplementary information, Figure S11 and Table S17). We also identified the expansion of NSY (neoxanthin synthase) and ABA4, which are involved in converting violaxanthin into neoxanthin, as well as the expansion of short-chain dehydrogenases/reductases (SDRs), which are candidate enzymes for converting xanthoxin into ABA aldehyde in the cytosol. The numbers of these genes are roughly two-fold those in other diploid plants, suggesting that the duplicated ABA *de novo* synthesis genes were retained after genome fusion in quinoa.

We also observed the expansion in the ABA receptor PYL family (Figure 3B, Supplementary information, Figure S12 and Table S17). Quinoa contains 22 PYL genes, while the other Caryophyllales contain at most 10. This number is also higher than potato, which contains 17 PYLs. Furthermore, quinoa contains a higher number of ABCGs, the group of ABC transporters that is utilized for ABA transportation in plant cells. These results suggest that the enhanced regulation of ABA homeostasis and signaling may contribute to abiotic stress tolerance in quinoa.

We examined the expression of ABA-responsive genes in different quinoa tissues. Among the 108 genes that are assigned the GO term “response to ABA stimulus”, 81% of them have a RPKM of at least three in any one of the seven tissues examined (Figure 3C) and 42% of them have a RPKM value of over 20 (Figure 3C), indicating that quinoa has a high basal level of ABA response.

### Transcriptome analyses of epidermal bladder cells

**Figure 4:**
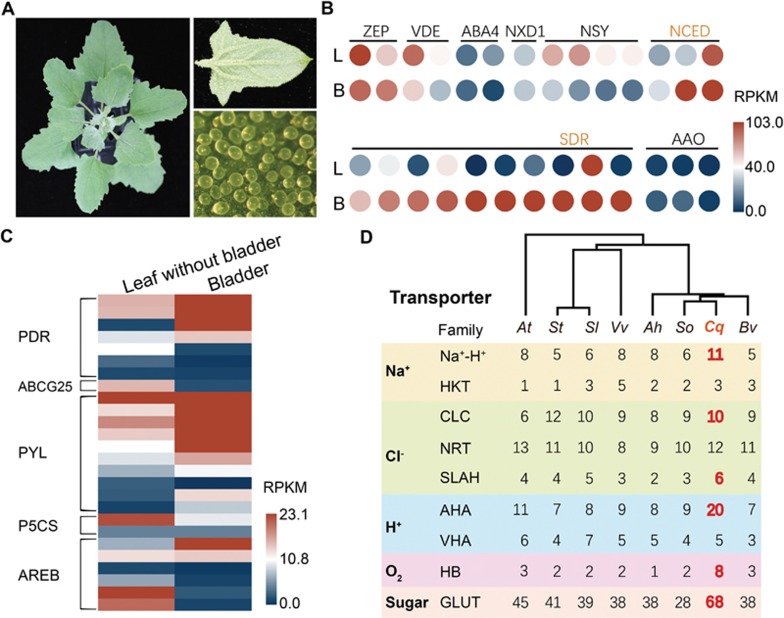
Prominent transporter and ABA biosynthesis activity in the epidermal bladder cells. **(A)** Photos of quinoa leaves and salt bladders. Left: top view of a 7-week-old quinoa plant. Right top: abaxial side of the fourth leaf of a 7-week-old quinoa plant; right bottom, epidermal bladder cells under a stereoscopic microscope, which are present at both the adaxial and abaxial leaf surfaces. **(B)** Transcript levels of ABA synthesis pathway genes in the leaf and the bladder cells. Only the genes that have a RPKM value higher than 1 in at least one tissue were presented. **(C)** Heatmap of the transcript level of genes involved in ABA transport and perception in the leaf and the bladder cells. **(D)** Copy number of genes that encode specific ion transporters, hemoglobin and sugar transporters mediating growth and stress response in quinoa and other plant species. Red numbers indicate gene copy numbers that are higher in quinoa. The phylogenetic relationship of these species is indicated at the top of the graph, inferred from orthologous gene sets. Species are indicated by two-letter names: *Ah*, *Amaranthus hypochondriacus*; *At*, *Arabidopsis thaliana*, *Bv*, sugar beet (*Beta vulgaris*), *Cq*, quinoa (*Chenopodium quinoa*), *Sl*, tomato (*Solanum lycopersicum*), *So*, spinach (*Spinacia oleracea L.*), *St*, potato (*Solanum tuberosum*), *Vv*, grape (*Vitis vinifera*).

Quinoa uses EBCs to sequester salts from metabolically active cells^34^ (Figure 4A). EBCs are observed in ∼50% of halophytic plants; so they are a common tissue utilized by salinity-tolerant plants to cope with salt. We thus performed transcriptome sequencing on bladder cells under salt-treated and non-treated conditions. Clustering analyses clearly showed that bladder cells exhibit a unique transcriptome profile among the different types of plant tissues tested, with a remote similarity to leaf cells (Supplementary information, Figure S13). Compared to a leaf without bladders, we identified 8 148 differentially expressed genes in bladder cells, consistent with EBCs being specialized cells for salt sequestration (Supplementary information, Figure S14). Among them, genes involved in the abiotic stress response, cell wall and suberin synthesis are enriched in upregulated genes, while genes involved in photosynthesis and genes encoding membrane proteins, especially chloroplast proteins, are enriched in downregulated genes, consistent with bladder cells being anabolically inactive salt sequesters (Supplementary information, Table S18). Considering the presumed high salt concentration (up to 1 M) in the vacuole of bladder cells and a pronounced salt gradient between the vacuole and cytoplasm, it has been proposed that a package of ion transporters is required to actively establish and maintain the gradient^3^. Among the genes that are more highly expressed in the bladder cells, we did identify a suite of ion transporters, including the anion transporters SLAH, NRT and ClC, the cation transporters NHX1 and HKT1 as well as the vacuolar and plasma membrane H^+^-ATPases, which generate proton gradients across the plasma and the vacuolar membranes to drive ion transport (Supplementary information, Figure S15). In addition to these ion transporters, the transcript level of more than 10 sugar transporters is higher in the bladder cells (Supplementary information, Figure S15). Considering the low photosynthetic activity of bladder cells, they are probably involved in transporting sugar into bladder cells to support their energy and nutrient needs. These results are consistent with the previous reports of EBCs maintaining highly negative membrane potentials (around −120 mV)^3^.

Enhanced transporter activity was not only detected at the transcript level but also at the gene level. Compared to other eudicot species, quinoa processes higher numbers of sodium-proton exchangers, HKTs (transporters for sodium and potassium) and SLAHs (Cl-permeable anion channels) (Figure 4D, Supplementary information, Table S19). The number of plasma membrane ATPases and sugar transporters also almost doubled in quinoa (Figure 4D, Supplementary information, Table S19), suggesting that during growth in saline environments, quinoa has evolved an elaborate system to maintain ion homeostasis throughout the plant.

After salt treatment, we identified 180 and 525 differentially expressed genes in leaf and bladder cells, respectively (Supplementary information, Figure S14). Only 25 genes were shared between the two groups, suggesting that leaf cells and bladder cells respond to salt treatment differently (Supplementary information, Figure S14). In the DEGs identified in bladder cells from salt-treated plants, genes involved in suberin and cutin biosynthesis are significantly enriched (Supplementary information, Table S18), suggesting that the wax content increases in response to elevated cellular salt concentrations. Interestingly, we found that one truncated hemoglobin (trHB) gene orthologous to the Arabidopsis *GLB3* was upregulated in EBCs after salt treatment (Supplementary information, Table S20). It also had a higher expression level in EBCs than in leaves (Supplementary information, Figure S15). The plant trHB was shown to have a moderate oxygen affinity^35^, so that it may be involved in promoting oxygen diffusion in the EBC. The family of hemoglobin genes also expanded in quinoa, which has eight members, compared to 1-3 in other plant species (Figure 4D). The majority of this family (7 out of 8) are orthologous to Arabidopsis HB1 or HB2, which are involved in nitric oxide (NO) metabolism (Supplementary information, Figure S16), suggesting that NO signaling may also be important for stress tolerance in quinoa.

Surprisingly, we found that the pathway of ABA *de novo* synthesis was upregulated in the bladder cells. The transcript levels of two NCED genes (CCG005786.3 and CCG042102.1), which are predicted to encode the rate-limiting enzyme for ABA biosynthesis, are 4-6-fold higher than in the leaves (Figure 4B). Some of the SDR genes that are homologous to Arabidopsis ABA2/SDR1 are more than 1 000-fold higher in bladder cells than in leaf cells (Figure 4B). Furthermore, in the bladder cells, we observed an elevated expression of ABA transporters, including ABCG40 (PDR) and ABCG25, as well as the majority of the PYL family of ABA receptor proteins (Figure 4C). These data suggest that bladder cells might maintain a high level of ABA homeostasis.

## Discussion

Despite a long history in studying the mechanisms of plant salinity tolerance, the engineering of salinity-tolerant crops remains very challenging. Salinity tolerance is a complex trait associated with multiple subtraits (e.g., ion homeostasis, osmotic balance and reactive oxygen species regulation), each having a complex genetic basis^36,37^. That makes traditional breeding for salt tolerance very difficult, if not impossible. Even with our understanding of a part of the underlying molecular pathways of the plant salt stress response and the identification of tens of quantitative trait loci associated with stress tolerance, the manipulation of a single or a limited number of genes has proved to be so far inadequate to create salinity-tolerant crops. This difficult situation can be alleviated in two ways. First, many of the glycophytic crops have close relatives in nature that are halophytes, and understanding the evolutionary trajectory of these species may shed light on how to engineer these crops. On the other hand, some of the minor crops usually cultivated in marginal areas are already salinity tolerant. Engineering these crops for a better yield could be an easier alternative than engineering major crops to be salt tolerant. Quinoa is a perfect candidate for both aspects and the genome assembly reported in this study could greatly facilitate the research in either direction.

The quinoa genome has a high content of repeat sequences, making up 64.5% of the genome, more than half of which are retrotransposons. The transposon amplification possibly happened after the divergence between *Amaranthus* and *Chenopodium* but before the genome fusion, as the genome size of quinoa fits the additive value of the A and B candidate parent genomes^15^.

We identified 54 438 protein-coding genes and functionally annotated 95.6% of them based on sequence homology. Quinoa genes belong to 14 707 orthologous groups with only 2.1% of them not identified in either *Arabidopsis* or three other *Amaranthaceae* species. Our analyses of the annotated quinoa genes indicated that enhanced transporter activities and increased ABA synthesis, transport and signaling may be critical for quinoa as a halophyte. Considering the independent evolution of halophytes in multiple plant lineages, it seems more likely that during evolution, plants found ways to better utilize their stress-fighting toolset than inventing new genes each time. This hypothesis is also supported by genomic and genetic studies in the salt crucifers *Eutrema salsugineum* and *Eutrema parvulum*, where an elevated basal level of stress responsiveness was found to be important in that knocking out the key players such as SOS1 eliminates the stress-tolerance ability^38,39,40,41,42^. Many of the ion transporters and ABA-related genes were expanded close to two-fold in quinoa, possibly a result of gene retention after genome duplication. This supports the notion that the WGD event is important for the evolution of salinity-tolerance traits in quinoa. However, polyploidy could increase or decrease salt tolerance^43^. To understand the genomic changes associated with the evolution of salinity tolerance, it will be interesting to compare quinoa to its diploid-relative species in the future.

The EBC is found in about half of the halophytes, while in the other half, salt is accumulated in metabolically inactive succulent tissues^3^. Our transcriptome analyses on this special cell type confirmed that it is a photosynthetically inactive tissue and has a strong activity in ion transportation, cell wall and wax synthesis. The latter may be used to generate a strong mechanical support and to prevent water loss. While the changes of the EBC transcriptome in response to salt treatment are consistent with the role of EBC in salt sequestration, the relatively small number of DEGs identified and the insignificant changes in the transcript level of most transporter genes suggest that bladder cells are constitutively active in salt sequestration (Supplementary information, Figure S15 and Table S18). We suggest that the regulation of ion transport in the bladder cell response to salt treatment may mainly occur at the protein level, e.g., through protein phosphorylation. Phosphorylation and dephosphorylation have been demonstrated to provide an on/off switch to the SLAC/SLAH anion channel, AKT Shaker-type K^+^ channels as well as SOS and HAK transporters^44,45,46^. The same transcriptome analyses also suggest that high ABA levels are required to maintain the cellular response to high osmotic stress within the bladder cell and ABA transporters are used for importing ABA from outside. Alternatively, bladder cells may function as an ABA-producing factory and export ABA to other parts of the plant for an elevated systematic response to abiotic stresses.

**Figure 5:**
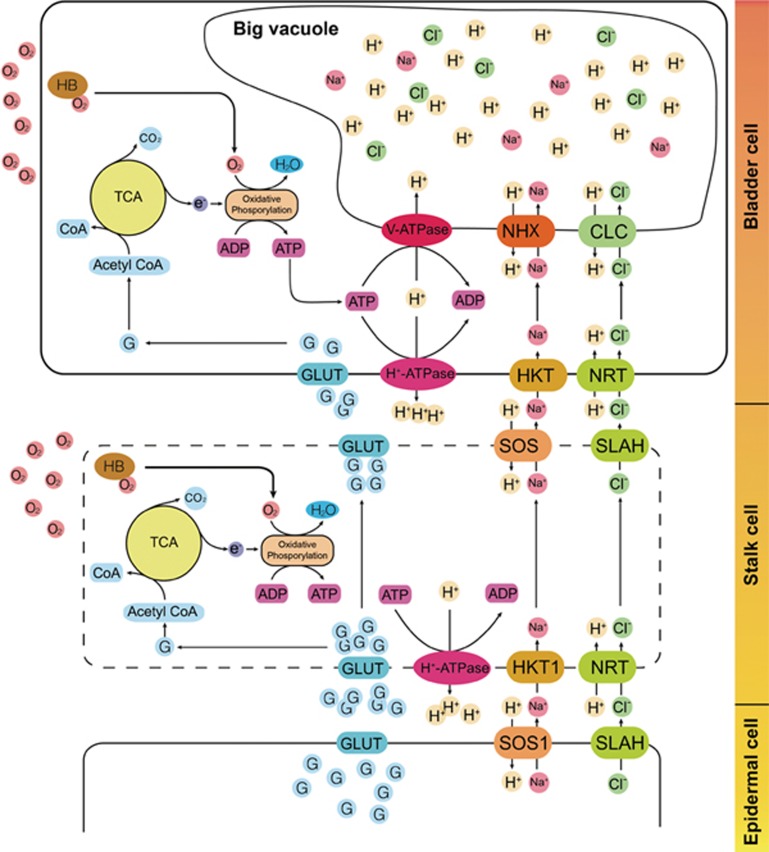
A proposed model for salt accumulation in bladder cells. Monosaccharides are transported from mesophyll cells to bladder cells by the GLUTs, and enter the TCA cycles for producing ATP. Hemoglobin (HB) helps with oxygen diffusion in stalk and bladder cells, and the oxygen is used for oxidative phosphorylation, which produces ATP. Vacuolar ATPase (V-ATPase) and plasma membrane H^+^ ATPase generate the proton gradients and membrane potential that are necessary for Na^+^ and Cl^−^ transport from the leaf to bladder cells, and then from the cytoplasm to the vacuole of bladder cells via Na^+^/H^+^ exchanger and chloride transporters, respectively. The ratio of cell sizes drawn in the diagram does not reflect the actual ratios. Stalk cells are represented in dashed lines because no transcriptome data are currently available for this type of cells.

Overall, the greater part of the sequence data has allowed us to modify the previously suggested model of salt accumulation in EBCs that is essential for plant adaptation to saline conditions^3^ (Figure 5). Toxic Na^+^ and Cl^−^ ions are expelled from the leaf mesophyll via SOS1 and SLAH, respectively, pass the stalk cell and are then loaded into EBC via HKT and NRT transporters, where they are sequestered in vacuoles by NHX and CLC. These transport processes are energized by plasma- and tonoplast-based H^+^ pumps. GLUT transporters enable the unidirectional movement of monosaccharides from the mesophyll into EBC to fuel these pumps, and hemoglobin (HB) facilitates oxygen diffusion into EBC to be used for oxidative phosphorylation and ATP production.

In future, it will be critical for quinoa research to develop transformation methods to facilitate gene manipulation. Our genome assembly will serve as a good starting point to access genetic diversities within the species, to guide targeted gene modification and for comparative genomics studies in Caryophyllales and halophytes.

## Materials and Methods

### Plant materials and growth conditions

*C. quinoa* f. *real* seeds were obtained from Bolivia and cultivated in the lab for four consecutive generations through single-seed descent. For genome sequencing, seeds from a single quinoa plant were germinated on the MS medium and grown in 1 L plastic pots using the ingredients described earlier^7^.

For mRNA sequencing, inbred quinoa plants were grown in a controlled greenhouse at a temperature between 19 °C and 26 °C and an average humidity of ∼65%. Dry seeds, 1-week-old seedlings and inflorescences, leaves and stems from 6-week-old plants were collected and flash frozen in liquid nitrogen. For mRNA sequencing in EBCs, *C. quinoa* (cv.5020) plants were cultivated in a climate chamber (12-h daylight, 50% rH, 20 °C) using a common potting soil. After 4 weeks, NaCl treatment was started. NaCl was added with the irrigation water for the salt-treated plants. Tap water was used for the control plants. The NaCl concentration in the irrigation water was increased stepwise up to 100 mM to prevent osmotic shock of root tissues. Plants were watered every second day. After 5 weeks of salt treatment, when the salt stress was visible by reduced growth of the plants, the plant tissue was harvested for RNA extraction.

### DNA extraction, library construction and sequencing

Genomic DNA was extracted using the Plant DNeasy Maxi Kit (Qiagen). For the two small-size libraries, genomic DNA was fragmented in a Covaris S220 and separated on a SAGE-ELF (Sage Science). Fractions from the SAGE-ELF that have an average size of approximately 380 bp and an average size of around 450 bp were used for PCR-free library construction using the TruSeq Nano DNA Library Preparation Kit (Illumina). The two medium-size libraries (5 kbp and 8 kbp) were prepared using the GS FLX Titanium Kit (Roche) following standard protocols. All the Illumina libraries were then sequenced on a HiSeq2500 at Core Facility for Genomics of Shanghai Center for Plant Stress Biology (PSC). The 20-kb PacBio library was prepared and sequenced at Tianjin Biochip Corporation, following the manufacturer's standard protocols.

### Genome assembly and assessment

In summary, three early assembly versions for the quinoa genome were generated using Illumina reads (v0.1) and PacBio reads (v0.2 and v0.3) separately. Then, the three assemblies were merged using the HABOT2 (hybrid assembly of third-generation sequencing 2; https://github.com/asarum/HABOT2) software (1gene Corp., Hangzhou, China). A final round of scaffolding and gap filling was performed using Illumina reads to obtain Cq_real_v1.0. A more detailed protocol can be found in Supplementary information, Data S1.

### Gene prediction, annotation and gene model assessment

We first annotated the repeats in Cq_real_v1.0 by combining *de novo* prediction and homology searches using RepeatMasker^47^. Then, the repetitive sequences were masked and three approaches were used for gene prediction: *ab initio* prediction, homology search and transcriptome-assisted gene prediction. Gene models derived from the three approaches were integrated to generate the final list of gene models using GLEAN^48^. A detailed description can be found in Supplementary information, Data S1.

We assessed the gene model in Cq_real_v1.0 for its completeness, sensitivity and specificity. Completeness was assessed by comparing the predicted protein sequences to the Pfam 31.0 database^49^ and to BUSCO v2^26^. Then, the sensitivity and specificity of the gene, exon and nucleotide level were evaluated using Eval v2.2.8^50^ by using mRNA sequences retrieved from the NCBI nucleotide database or high-expression genes assembled from mRNA-seq data. A detailed description can be found in Supplementary information, Data S1.

### Phylogenetic analysis

OrthoFinder was used to determine the genes that were orthologous among seven species (*A. thaliana*, *C. quinoa*, *Oryza sativa*, *Vitis vinifera*, *A. hypochondriacus*, *B. vulgaris* and *S. oleracea*)^51^. All-versus-all BLASTP with an E-value cutoff of 1e−05 was performed and orthologous genes were clustered using OrthoFinder. Single-copy orthologous genes were extracted from the clustering results using an in-house script. MAFFT v7 was used to perform multiple-sequence alignment for each group of single-copy orthologous genes with default parameters, and transform the protein sequence alignments into nucleotide sequence alignments^52^. Conserved blocks were extracted from the multiple-sequence alignment results using Gblocks 0.91^53^ (a minimum number of sequences for a conserved position: 9; a minimum number of sequences for a flank position: 14; a maximum number of contiguous nonconserved positions: 8 and a minimum length of a block: 10), and then concatenated to one supergene for phylogenetic analysis. PhyML3.0^54^ was used to build the species phylogenetic tree with the parameters: -m HKY85-rates gamma-b-4, and PAML v4.8a^55^ was used to compute the mean substitution rates and to estimate the species divergence time.

### Colinearity analyses

For gene colinearity analyses, all-versus-all BLASTP searches (with an E-value cutoff of 1 × 10^−5^) were performed to identify paralogous or orthologous gene pairs. Colinear blocks containing at least five genes were identified using MCScanX with the parameters: -s 3 -m 5^56^. The *K*_a_ and *K*_s_ value of paralogous or orthologous gene pairs was calculated using an in-house Perl script. Circos software^57^ was used to illustrate the position relationships between syntenic blocks on the assembled scaffolds.

### Gene copy analyses

A well-characterized *Arabidopsis* member of each protein family was used as a seed to search for similar protein sequences in quinoa and other plant species using BLASTP. Candidates with an E-value smaller than 10e−5 were then searched against the Pfam 31.0 database^49^ to ensure that all the major conserved domains that are present in the seed are also present in the candidates. The candidate protein sequences were then manually examined to exclude the outliers that have very short protein sequences relative to others. The gene copy number in each species is manually counted.

### RNA extraction and library preparation

RNA from salt bladders was isolated using the NucleoSpin RNA Plant Kit (Macherey-Nagel) according to the manual with the following modifications. Young leaves having mostly intact, turgescent bladders were dipped into liquid nitrogen. The bladders were scratched off from the leaves with a small spatula. For each sample, bladders of six young leaves from two individual plants were collected in 500 μl of lysis buffer RA1 (containing 1:100 TCEP) and mixed vigorously for 20 s to lyse the cells. The lysate was transferred to NucleoSpin Filters and centrifuged for 1 min at 11 000× *g*. The filtrate was mixed with 500 μl of ethanol (70%) and transferred in two steps to a NucleoSpin RNA plant Column and centrifuged for 1 min at 11 000× *g*. The washing steps were performed following the manufacturer's instructions. RNA was eluted in 33 μl of RNase-free water that was incubated on the membrane for 1 min, centrifuged and incubated for a second time on the membrane. RNA from leaves as well as leaves without bladders was isolated using the E.Z.N.A plant RNA Kit (OMEGA Bio-Tek) following the manufacturer's instructions. The leaves were of the same age as those from which the bladders were removed. The rest of the latter was used for the “leaves without bladder” samples. Strand-specific mRNA libraries were prepared at Core Facility for Genomics at Shanghai Center for Plant Stress Biology (PSC) using NEBNext Ultra Directional RNA Library Prep Kit for Illumina (New England BioLabs, Cat No. E7420). The libraries were then sequenced on a HiSeq2500 (Illumina) using the paired-end 125-bp sequencing mode.

### Transcriptome analyses

Raw sequencing reads were filtered using a quality cutoff of 30 while removing the adapter sequences. The clean reads were then mapped to the Cq_real_v1.0 assembly using subread-align (v1.5.1)^58^. Only uniquely mapped paired-end reads were retained for read counting against the annotated gene models using featureCounts (v1.5.1)^59^. The edgeR package (v3.14.0) was then used to identify differentially expressed genes.

### Data availability

The genome sequence and gene models of Cq_real_v1.0 are available at the NCBI genome database with the BioProject number PRJNA394587. The high-throughput sequencing data for genome assembly, developmental transcriptome and EBC transcriptome analyses are available at Sequence Read Archive (https://www.ncbi.nlm.nih.gov/sra) under the BioProject number PRJNA394587, PRJNA394651 and PRJNA394652, respectively. The source code for HABOT2 is available at GitHub (https://github.com/asarum/HABOT2).

## Author Contributions

CZ, AC, LX, HMM, PA, MZ, WJ, PD, RH, XW and JB performed the experiments, CZ, GH, DL, DZ, FL and RL performed data analyses, HuiZ, SS, RH, JKZ and HZ designed the experiments and CZ, SS, RH, JKZ and HZ wrote the paper.

## Competing Financial Interests

The authors declare no competing financial interests.

## Figures and Tables

**Table 1:** Global statistics of *Chenopodium quinoa* genome assembly and annotation

	Number	Size
***Assembly feature***		
Estimated genome size		1 482 Mb
Total scaffolds (≥ 100 bp)	3 184	1 337 Mb
Scaffold N50	373	1 162 Kb
Longest scaffold		5 398 Kb
Total contigs	10 795	1 324 Mb
Contig N50	1 536	268 Kb
Longest contig		1 594 Kb
GC content	37.0%	
***Genome annotation***		
Total repetitive sequences	64.5%	863 Mb
Transposable elements	55.3%	739 Mb
Gene models	54 438	201 Mb
Noncoding RNAs	10 358	1 083 Kb

## Supplementary Material

Supplementary information, Figure S1 The distribution of 17-kmer frequencies of *C. quinoa*.

Supplementary information, Figure S2 The chloroplast genome of *C. quinoa*.

Supplementary information, Figure S3 Examples of Cq_real_v1.0 scaffolds that are anchored to a published genetic map of quinoa (Maughan et al. 2012).

Supplementary information, Figure S4 Assessment of gene prediction in Cq_real_v1.0.

Supplementary information, Figure S5 Scatterplot showing the RPKM values of the 10,554 genes that are predicted in Cq_real_v1.0 but not in ASM168347v1.

Supplementary information, Figure S6 Venn diagram showing the overlap of orthologous groups among quinoa and 4 other plant species.

Supplementary information, Figure S7 Histogram of Ks (synonymous substitution) values for paralogous quinoa gene pairs.

Supplementary information, Figure S8 Composition of 19 amino acid residues (except Lys) in albumin proteins in five different crops.

Supplementary information, Figure S9 Composition of 19 amino acid residues (except Lys) in globulin proteins in five different crops.

Supplementary information, Figure S10 Composition of 19 amino acid residues (except Lys) in LEA proteins in five different crops.

Supplementary information, Figure S11 Unrooted phylogenetic tree of NCED genes.

Supplementary information, Figure S12 Unrooted phylogenetic tree of PYL genes.

Supplementary information, Figure S13 Matrix of Pearson's correlation coefficients based on RPKM values of all annotated genes.

Supplementary information, Figure S14 Venn diagram showing the overlap of DEGs (differentially expressed genes) among the four indicated comparisons.

Supplementary information, Figure S15 Heatmap showing the RPKM value of the transporter genes for ions, monosaccharides and ABA in leaf without bladders and bladder cells.

Supplementary information, Figure S16 Unrooted phylogenetic tree of hemoglobin genes.

Supplementary information, Table S1 Summary of sequencing data used for de novo assembly

Supplementary information, Table S2 Summary of different Cq assembly versions

Supplementary information, Table S3 Summary of gene space assessment using de novo assembled EST sequences

Supplementary information, Table S4 Summary of fosmid alignment with published quinoa assemblies

Supplementary information, Table S5 Summary of transposable elements (TEs) in Cq_real_v1.0

Supplementary information, Table S6 Summary of SSRs (simple sequence repeats) identified in quinoa

Supplementary information, Table S7 A list of top SSRs identified in quinoa

Supplementary information, Table S8 Statistics of predicted quinoa gene models at each step of the annotation pipeline

Supplementary information, Table S9 Statistics of noncoding RNAs (ncRNA) identified in the quinoa genome

Supplementary information, Table S10 Summary of quinoa genes with assigned (predicted) functions

Supplementary information, Table S11 Summary of Cq genes identified in BUSCO v2 embryophyta gene set

Supplementary information, Table S12 Estimated accuracy of gene prediction

Supplementary information, Table S13 Comparison of Cq_real_v1.0 with two published quinoa assemblies

Supplementary information, Table S14 Enriched GO terms in quinoa-specific orthologous groups

Supplementary information, Table S15 List of other genomes used in this study

Supplementary information, Table S16 List of seed storage proteins in quinoa and 4 other crops, related to Figure 2H

Supplementary information, Table S17 List of ABA synthesis and signaling related proteins in quinoa and 7 other species, related to Figure 3B

Supplementary information, Table S18 Differentially expressed genes and enriched GO terms by comparing EBCs and leaves

Supplementary information, Table S19 List of transporter proteins in quinoa and 7 other species, related to Figure 4D

Supplementary information, Table S20 Expression levels of transporter genes in different quinoa tissue
