# Supplementary material for: A high-quality genome assembly of quinoa provides insights into the molecular basis of salt bladder-based salinity tolerance and the exceptional nutritional value
Source: Cell Res. 2017 Oct 10;27(11):1327–40. doi: 10.1038/cr.2017.124 (PMC5674158; doi:10.1038/cr.2017.124)
Supplement: Supplementary information, Figure S1 — The distribution of 17-kmer frequencies of C. quinoa. [file cr2017124x1.pdf]

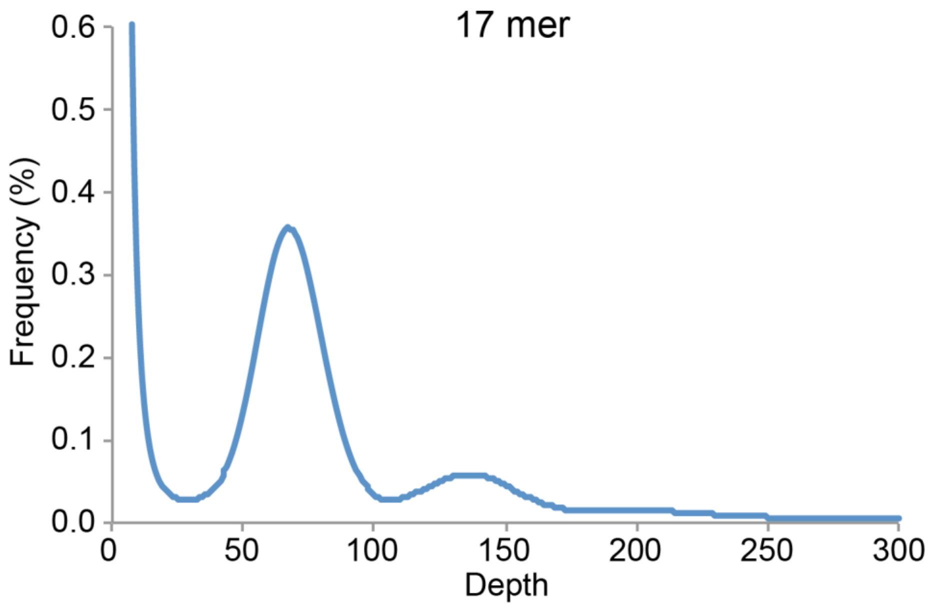

Supplementary information, Figure S1 The distribution of 17-kmer frequencies of *C. quinoa*.

The frequency of 17-mers peaks at 67. The genome size is estimated using the formula: Genome size = K-mer number/peak depth. A total of 344,467,594 reads from the two PCR-free libraries were used, which resulted in 99,297,839,479 K-mers.

K-mer Number	Peak Depth	Genome Size (nt)	Used Bases	Used Reads	Depth
99,297,839,479	67	1,482,057,305	86,461,366,094	344,467,594	58.4
