# Supplementary material for: A high-quality genome assembly of quinoa provides insights into the molecular basis of salt bladder-based salinity tolerance and the exceptional nutritional value
Source: Cell Res. 2017 Oct 10;27(11):1327–40. doi: 10.1038/cr.2017.124 (PMC5674158; doi:10.1038/cr.2017.124)
Supplement: Supplementary information, Figure S2 — The chloroplast genome of C. quinoa. [file cr2017124x2.pdf]

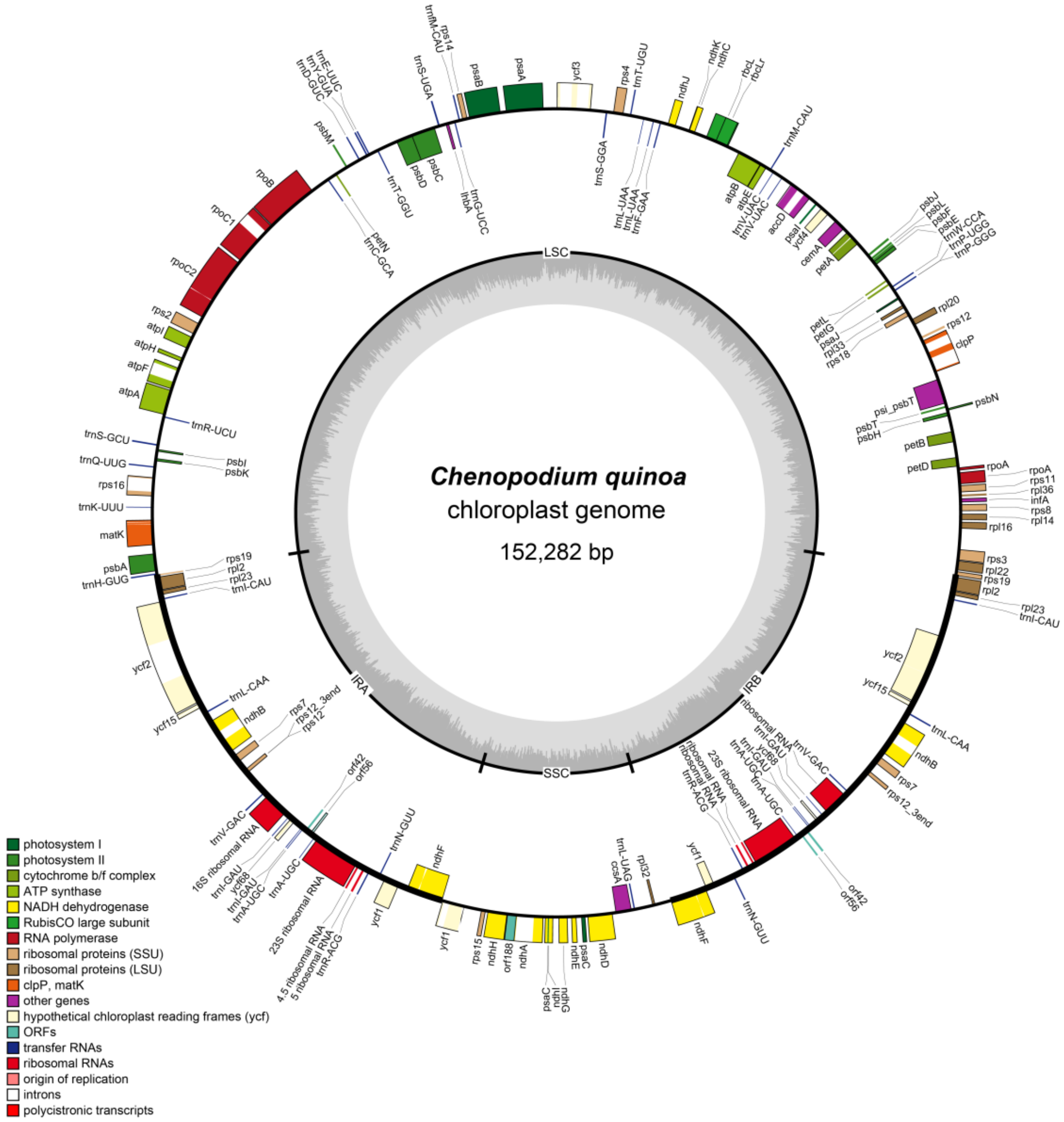


**Supplementary information, Figure S2** The chloroplast genome of *C. quinoa*.

The bold inner circle indicates the 4 conserved regions of the chloroplast genome: two inverted repeats (IRA and IRB) that separate a short single copy (SSC) region and a long single copy (LSC) region. The inner dark grey bars show the coverage of the chloroplast genome in non-overlapping 10-bp window. Genes located on the (+) and (-) strand are indicated on the outside and inside of the circle respectively.
