# Supplementary material for: A high-quality genome assembly of quinoa provides insights into the molecular basis of salt bladder-based salinity tolerance and the exceptional nutritional value
Source: Cell Res. 2017 Oct 10;27(11):1327–40. doi: 10.1038/cr.2017.124 (PMC5674158; doi:10.1038/cr.2017.124)
Supplement: Supplementary information, Figure S3 — Examples of Cq_real_v1.0 scaffolds that are anchored to a published genetic map of quinoa (Maughan et al. 2012). [file cr2017124x3.pdf]

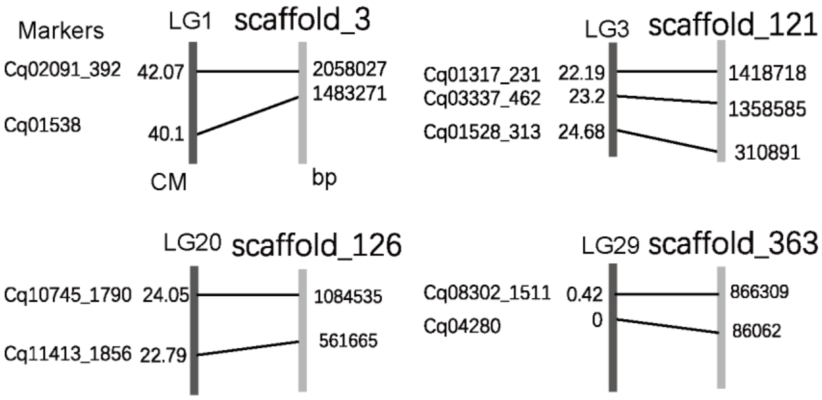

Supplementary information, Figure S3 Examples of Cq_real_v1.0 scaffolds that are anchored to a published genetic map of quinoa (Maughan et al. 2012).

Names of the SNP markers and their positions on different linkage groups (LG) are indicated on the left and the corresponding scaffolds with the position (bp) of SNP markers are indicated on the right.
