# Supplementary material for: A high-quality genome assembly of quinoa provides insights into the molecular basis of salt bladder-based salinity tolerance and the exceptional nutritional value
Source: Cell Res. 2017 Oct 10;27(11):1327–40. doi: 10.1038/cr.2017.124 (PMC5674158; doi:10.1038/cr.2017.124)
Supplement: Supplementary information, Figure S4 — Assessment of gene prediction in Cq_real_v1.0. [file cr2017124x4.pdf]

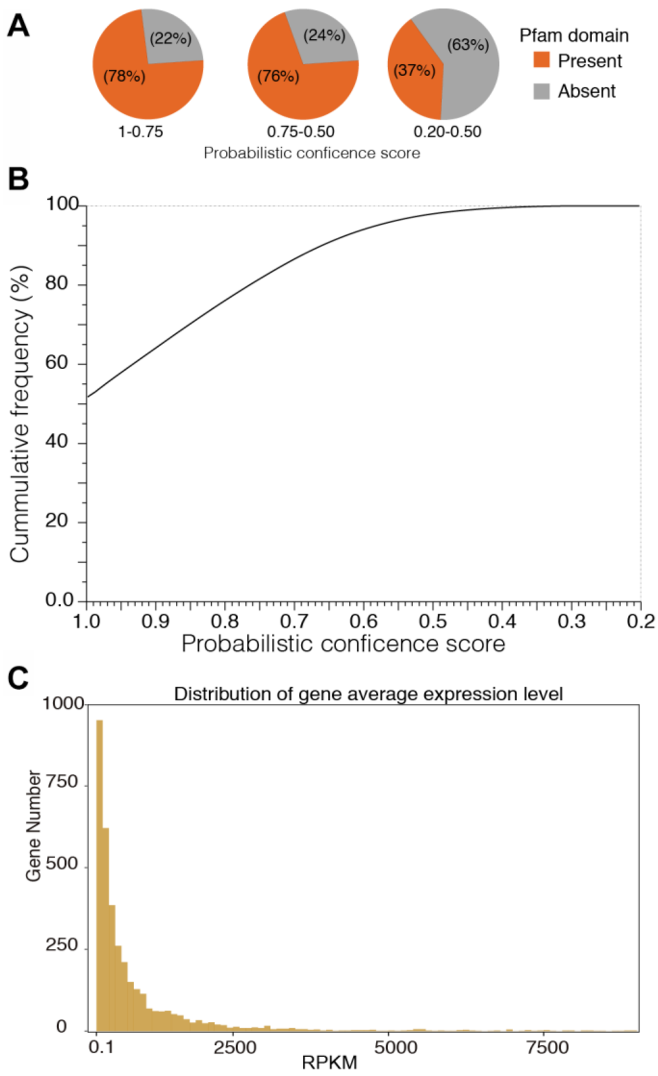

Supplementary information, Figure S4 Assessment of gene prediction in Cq_real_v1.0.

(A) Cumulative frequency of the probabilistic confidence score of predicted genes in Cq_real_v1.0 given by GLEAN. (B) Pie charts showing the percentage of gene sets that have conserved protein domains present or absent in the protein sequence. Conserved domains were identified by blast against the Pfam database using a E-value cutoff of 1E-4. (C) Distribution of RPKM values of all the annotated genes. The mRNA-seq data from 5 different types of quinoa tissue were used.
