# Supplementary material for: A high-quality genome assembly of quinoa provides insights into the molecular basis of salt bladder-based salinity tolerance and the exceptional nutritional value
Source: Cell Res. 2017 Oct 10;27(11):1327–40. doi: 10.1038/cr.2017.124 (PMC5674158; doi:10.1038/cr.2017.124)
Supplement: Supplementary information, Figure S5 — Scatterplot showing the RPKM values of the 10,554 genes that are predicted in Cq_real_v1.0 but not in ASM168347v1. [file cr2017124x5.pdf]

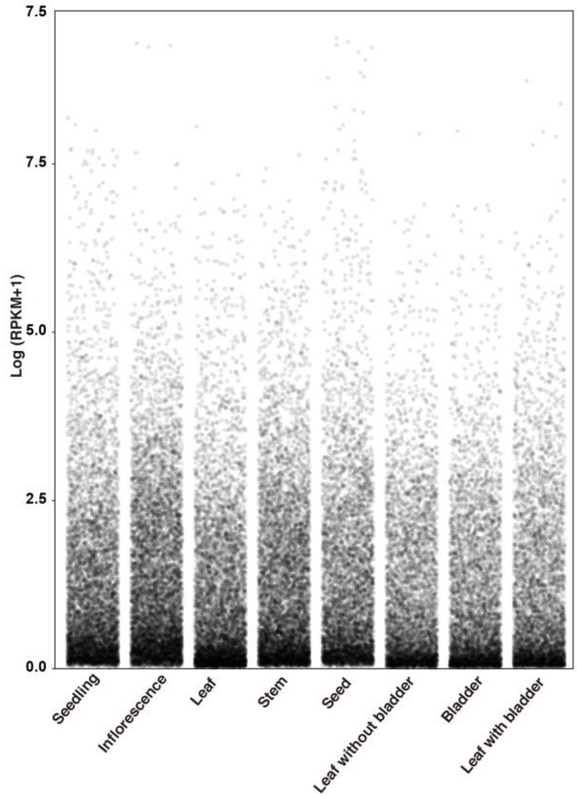

Supplementary information, Figure S5 Scatterplot showing the RPKM values of the 10,554 genes that are predicted in Cq_real_v1.0 but not in ASM168347v1.

The 8 different types of quinoa tissue used for mRNA-seq are indicated in the x-axis and the log transformed RPKM value are in the y-axis.
