# Supplementary material for: A high-quality genome assembly of quinoa provides insights into the molecular basis of salt bladder-based salinity tolerance and the exceptional nutritional value
Source: Cell Res. 2017 Oct 10;27(11):1327–40. doi: 10.1038/cr.2017.124 (PMC5674158; doi:10.1038/cr.2017.124)
Supplement: Supplementary information, Figure S6 — Venn diagram showing the overlap of orthologous groups among quinoa and 4 other plant species. [file cr2017124x6.pdf]

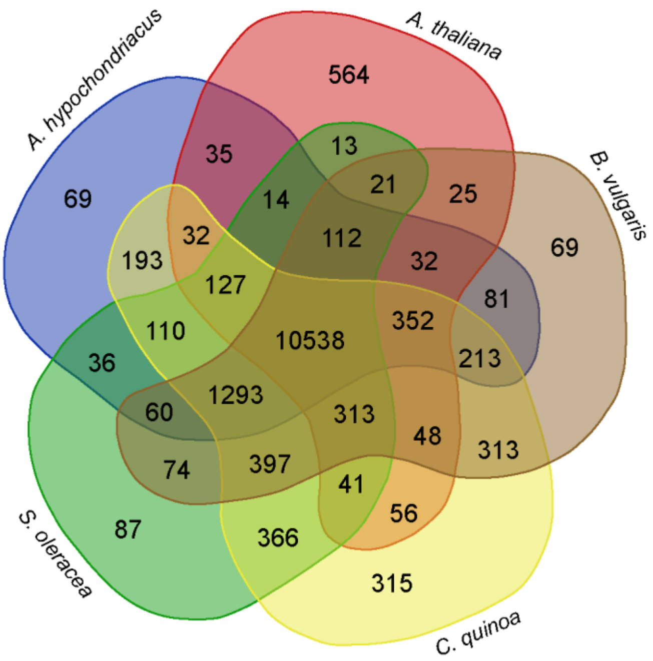

Supplementary information, Figure S6 Venn diagram showing the overlap of orthologous groups among quinoa and 4 other plant species.
