# Supplementary material for: A high-quality genome assembly of quinoa provides insights into the molecular basis of salt bladder-based salinity tolerance and the exceptional nutritional value
Source: Cell Res. 2017 Oct 10;27(11):1327–40. doi: 10.1038/cr.2017.124 (PMC5674158; doi:10.1038/cr.2017.124)
Supplement: Supplementary information, Figure S10 — Composition of 19 amino acid residues (except Lys) in LEA proteins in five different crops. [file cr2017124x10.pdf]

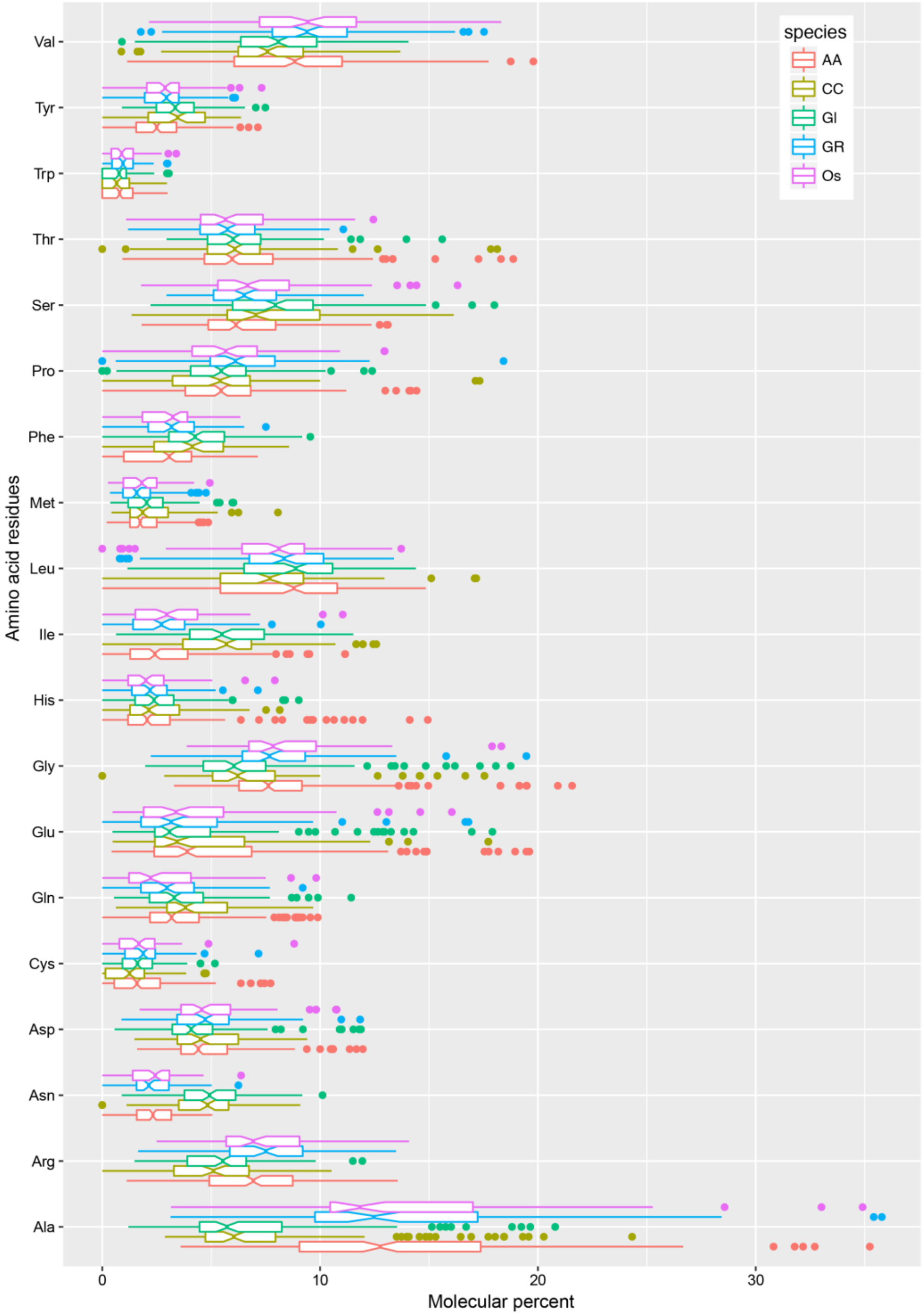

Supplementary information, Figure S10 Composition of 19 amino acid residues (except Lys) in LEA proteins in five different crops.

AA, *Aegilops tauschii*; CC, *Chenopodium quinoa*; Gl, *Glycine max*; GR, *Zea mays*; Os, *Oryza sativa*.
