# Supplementary material for: A high-quality genome assembly of quinoa provides insights into the molecular basis of salt bladder-based salinity tolerance and the exceptional nutritional value
Source: Cell Res. 2017 Oct 10;27(11):1327–40. doi: 10.1038/cr.2017.124 (PMC5674158; doi:10.1038/cr.2017.124)
Supplement: Supplementary information, Figure S12 — Unrooted phylogenetic tree of PYL genes. [file cr2017124x12.pdf]

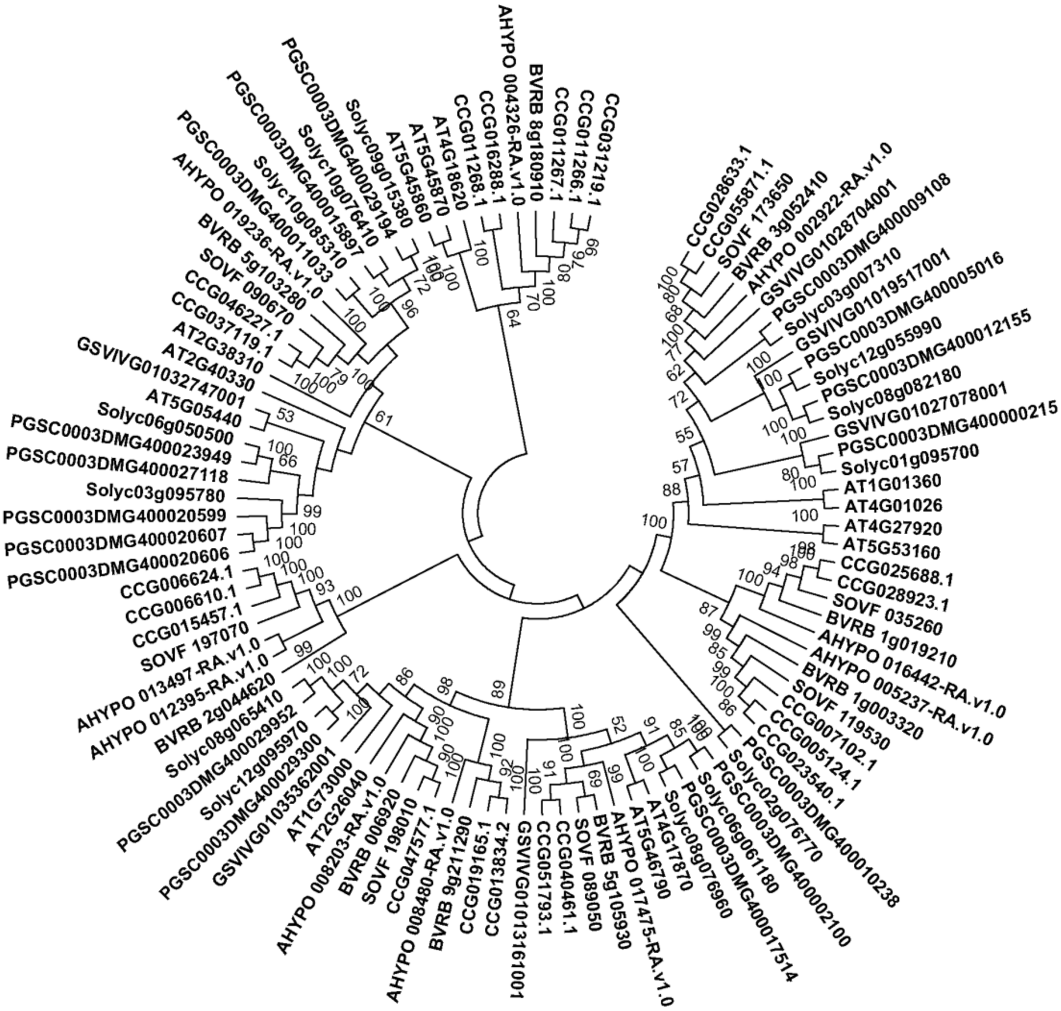

Supplementary information, Figure S12 Unrooted phylogenetic tree of PYL genes. The consensus tree was generated based a multiple alignment of the CDS (coding sequence) of PYL genes from quinoa and 7 other plant species as in Figure 3B. The codon alignment was generated by Muscle in MEGA7.0 and the tree was constructed using the neighbor-joining method with 1000 bootstrap replicates. Numbers at each branching point indicate that the local bootstrap values per 100 replicates. The origin of genes can be distinguished based on the first few letters of the gene name (*A. hypochondriacus* - AHYPO*, A. thaliana* – AT*; S. oleracea* - SOVF*, B. vulgaris* - BVRB*, V. vinifera* - GSVIVG*, S. lycopersicum*- Solyc and *S. tuberosum* – PGSC*, C. quinoa* - CCG).
