# Supplementary material for: A high-quality genome assembly of quinoa provides insights into the molecular basis of salt bladder-based salinity tolerance and the exceptional nutritional value
Source: Cell Res. 2017 Oct 10;27(11):1327–40. doi: 10.1038/cr.2017.124 (PMC5674158; doi:10.1038/cr.2017.124)
Supplement: Supplementary information, Figure S13 — Matrix of Pearson's correlation coefficients based on RPKM values of all annotated genes. [file cr2017124x13.pdf]

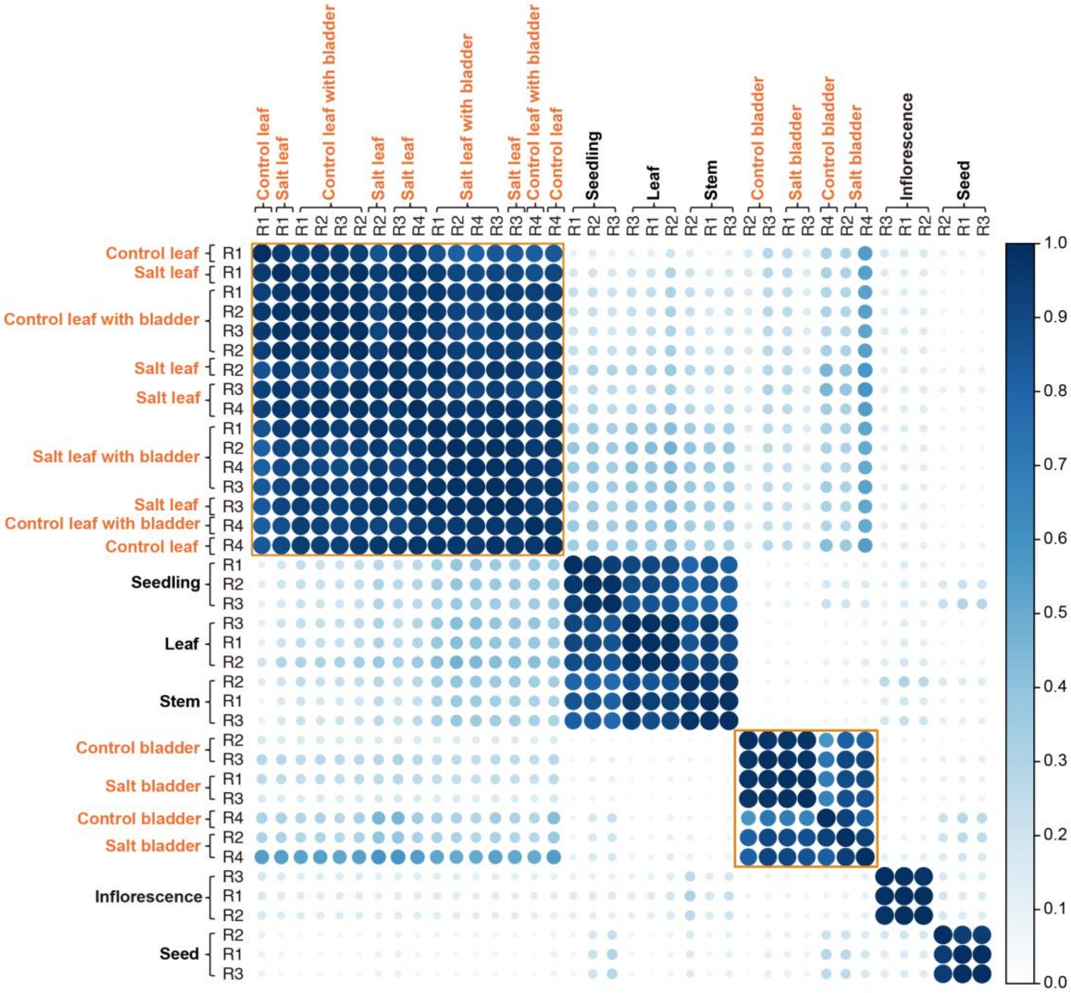

Supplementary information, Figure S13 Matrix of Pearson's correlation coefficients based on RPKM values of all annotated genes.

R1, R2, R3 and R4 represented the biological replicates of each indicated tissue type. The size of the dot and the depth of blue color positively correlate with the coefficient on a scale from 0 to 1.
