# Supplementary material for: A high-quality genome assembly of quinoa provides insights into the molecular basis of salt bladder-based salinity tolerance and the exceptional nutritional value
Source: Cell Res. 2017 Oct 10;27(11):1327–40. doi: 10.1038/cr.2017.124 (PMC5674158; doi:10.1038/cr.2017.124)
Supplement: Supplementary information, Figure S15 — Heatmap showing the RPKM value of the transporter genes for ions, monosaccharides and ABA in leaf without bladders and bladder cells. [file cr2017124x15.pdf]

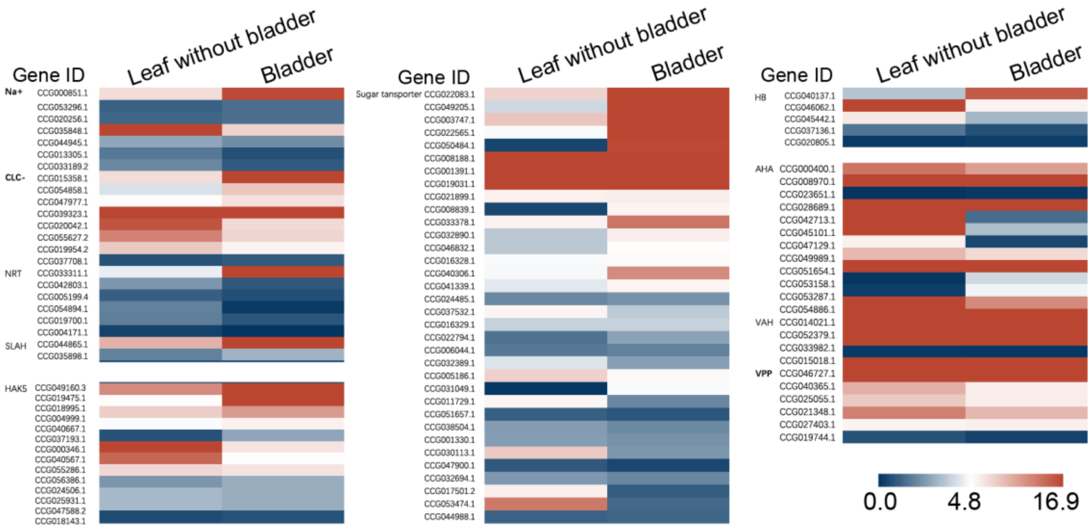

Supplementary information, Figure S15 Heatmap showing the RPKM value of the transporter genes for ions, monosaccharides and ABA in leaf without bladders and bladder cells.

The category of genes and the gene ID are indicated to the left of the heatmap. The legend indicates the correlation between colors and RPKM values.
