# Supplementary material for: A high-quality genome assembly of quinoa provides insights into the molecular basis of salt bladder-based salinity tolerance and the exceptional nutritional value
Source: Cell Res. 2017 Oct 10;27(11):1327–40. doi: 10.1038/cr.2017.124 (PMC5674158; doi:10.1038/cr.2017.124)
Supplement: Supplementary information, Figure S16 — Unrooted phylogenetic tree of hemoglobin genes. [file cr2017124x16.pdf]

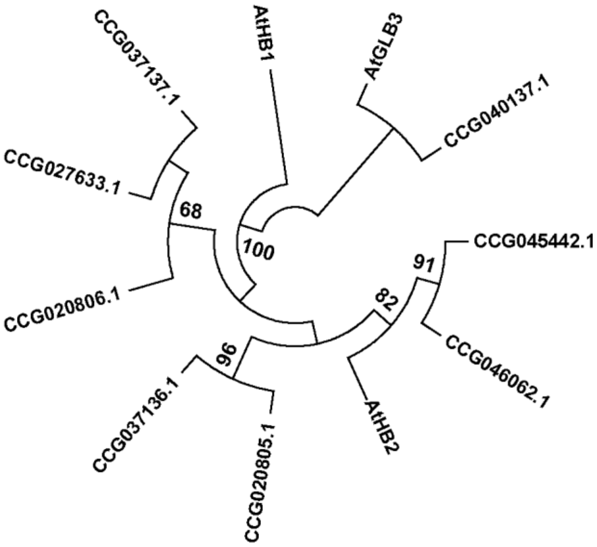

Supplementary information, Figure S16 Unrooted phylogenetic tree of hemoglobin genes.

The consensus tree was generated based a multiple alignment of the CDS (coding sequence) from quinoa and Arabidopsis thalian. The codon alignment was generated by Muscle in MEGA7.0 and the tree was constructed using the neighbor-joining method with 1000 bootstrap replicates. Numbers at each branching point indicate that the local bootstrap values per 100 replicates.
