# Supplementary material for: A high-quality genome assembly of quinoa provides insights into the molecular basis of salt bladder-based salinity tolerance and the exceptional nutritional value
Source: Cell Res. 2017 Oct 10;27(11):1327–40. doi: 10.1038/cr.2017.124 (PMC5674158; doi:10.1038/cr.2017.124)
Supplement: Supplementary information, Table S1 — Summary of sequencing data used for de novo assembly [file cr2017124x17.pdf]

Supplementary Table 1. Summary of sequencing data used for de novo assembly

Raw	Library No. (Illumina)	Insert Size	Amount of Data (Gb)	Read Length (bp)	Sequence Depth (x)	Physical Depth (x)
	1	380 bp	27.97	250_250	18.87	14.34
	2	380 bp	90.58	250_250	61.15	46.45
	3	5 kb	20.75	250_250	14.00	140.01
			24.62	125_125	16.61	332.25
	4	8 kb	38.24	125_125	25.80	825.70
			18.37	250_250	12.40	198.33
	5 (PacBio)	20 kb	50.3	7,521 (average)	33.94	33.94
	Total		213.97		182.77	1591.02

Filtered	Library No. (Illumina)	Insert Size	Amount of Data (Gb)	Read Length (bp)	Sequence Depth (x)	Physical Depth (x)
	1	380 bp	23.7	250_250	16.01	9.37
	2	380 bp	62.76	250_250	42.41	31.44
	3	5 kb	2.99	50_50	2.02	100.88
	4	8 kb	4.79	50_50	3.23	258.57
	5 (PacBio)	20 kb	50.3	7,521 (average)	33.94	33.94
	Total		153.41		97.61	434.20
