# Supplementary material for: A high-quality genome assembly of quinoa provides insights into the molecular basis of salt bladder-based salinity tolerance and the exceptional nutritional value
Source: Cell Res. 2017 Oct 10;27(11):1327–40. doi: 10.1038/cr.2017.124 (PMC5674158; doi:10.1038/cr.2017.124)
Supplement: Supplementary information, Table S2 — Summary of different Cq assembly versions [file cr2017124x18.pdf]

Table S2. Summary of different Cq assembly versions

v0.1	Scaffold		Contig	
	Size (bp)	Number	Size (bp)	Number
N90	284	444,927	269	482,414
N80	736	92,438	667	126,648
N70	13,363	18,162	6,374	34,478
N60	32,172	11,290	16,613	21,015
N50	49,570	7,592	26,127	14,095
Longest	499,594	----	281,357	----
Total Size	1,490,886,271	----	1,450,125,437	----
Total Number (≥100bp)		1,142,478	----	1,165,224
Total Number (≥2kb)		33,071	----	52,781

v0.2	Contig	
	Size (bp)	Number
N90	14,775	14,393
N80	23,994	10,451
N70	33,267	7,801
N60	43,470	5,827
N50	53,964	4,281
Longest	365,916	----
Total Size	750,878,283	----
Total Number (≥100bp)	----	25,024
Total Number (≥2kb)	----	23,874

v0.3	Scaffold		Contig	
	Size (bp)	Number	Size (bp)	Number
N90	9,609	37,102	9,606	37,107
N80	13,389	24,304	13,388	24,306
N70	22,513	15,803	22,509	15,804
N60	36,585	10,814	36,582	10,815
N50	51,182	7,493	51,182	7,493
Longest	586,601	----	586,601	----
Total Size	1,439,881,532	----	1,439,878,528	----
Total Number (≥100bp)		59,594	----	59,618
Total Number (≥2kb)		58,870	----	58,881

v1.0	Scaffold		Contig	
	Size (bp)	Number	Size (bp)	Number
N90	422,582	1,087	75,678	5,001
N80	629,431	833	125,667	3,657
N70	810,545	647	172,908	2,761
N60	985,512	498	220,272	2,082
N50	1,161,829	373	268,320	1,536
Longest	5,397,643	----	1,594,973	----
Total Size	1,337,226,356	----	1,325,521,993	----
Total Number (≥100bp)		3,184		10,795
Total Number (≥2kb)		2,827		9,981
