# Supplementary material for: A high-quality genome assembly of quinoa provides insights into the molecular basis of salt bladder-based salinity tolerance and the exceptional nutritional value
Source: Cell Res. 2017 Oct 10;27(11):1327–40. doi: 10.1038/cr.2017.124 (PMC5674158; doi:10.1038/cr.2017.124)
Supplement: Supplementary information, Table S3 — Summary of gene space assessment using de novo assembled EST sequences [file cr2017124x19.pdf]

Table S3. Summary of gene space assessment using de novo assembled EST sequences

	Number	Total length (bp)	Covered by assembly (%)	with >90% sequence in one scaffold		with >50% sequence in one scaffold	
				Number	Percent (%)	Number	Percent (%)
All	234,311	264,398,310	98.938	223,625	95.439	234,149	99.931
>200bp	234,311	264,398,310	98.938	223,625	95.439	234,149	99.931
>500bp	151,776	237,088,802	98.903	144,416	95.151	151,672	99.931
>1000bp	98,860	198,932,903	98.970	94,075	95.160	98,798	99.937
