# Supplementary material for: A high-quality genome assembly of quinoa provides insights into the molecular basis of salt bladder-based salinity tolerance and the exceptional nutritional value
Source: Cell Res. 2017 Oct 10;27(11):1327–40. doi: 10.1038/cr.2017.124 (PMC5674158; doi:10.1038/cr.2017.124)
Supplement: Supplementary information, Table S4 — Summary of fosmid alignment with published quinoa assemblies [file cr2017124x20.pdf]

Table S4. Summary of fosmid alignment with published quinoa assemblies

Fosmids		Cq_real_v1.0 (This study)					ASM168347v1 (Jarvis et al. 2017)				
**Fosmid ID**	**length**	**Target scaffold**	**Target match**	**score**	**mismatch**	**gap**	**Target scaffold**	**Target match**	**score**	**mismatch**	**gap**
fosmid1	31691	scaffold_0031	31856	31681	4	171	C_Quinoa_Scaffold_3525	31864	31668	9	187
fosmid2	31421	scaffold_0094	31877	31277	102	498	C_Quinoa_Scaffold_1319	31732	31260	123	349
fosmid3	33944	scaffold_0440	34642	33796	83	763	C_Quinoa_Scaffold_3901	34221	33656	111	454
fosmid4	33082	scaffold_0742	33267	33026	8	233	C_Quinoa_Scaffold_4480	33359	33022	30	307
fosmid5	38339	scaffold_0103	38545	38280	29	236	C_Quinoa_Scaffold_3144	38614	38249	62	303
fosmid6	30450	scaffold_0165	30604	30333	57	214	C_Quinoa_Scaffold_3820	30615	30125	90	400
fosmid7	33655	scaffold_0122	33858	33607	14	237	C_Quinoa_Scaffold_3298	33898	33605	22	271
fosmid8	36137	scaffold_0270	36334	35922	82	330	C_Quinoa_Scaffold_1465	36772	35800	171	801
fosmid9	30327	scaffold_0351	30527	30193	85	249	C_Quinoa_Scaffold_3388	28062	27735	84	243
fosmid10	32749	scaffold_0949	32848	32722	1	125	C_Quinoa_Scaffold_3107	33245	32664	27	554
