# Supplementary material for: A high-quality genome assembly of quinoa provides insights into the molecular basis of salt bladder-based salinity tolerance and the exceptional nutritional value
Source: Cell Res. 2017 Oct 10;27(11):1327–40. doi: 10.1038/cr.2017.124 (PMC5674158; doi:10.1038/cr.2017.124)
Supplement: Supplementary information, Table S5 — Summary of transposable elements (TEs) in Cq_real_v1.0 [file cr2017124x21.pdf]

Table S5. Summary of transposable elements (TEs) in Cq_real_v1.0

Superfamily of TEs	Coverage of TEs (bp)	Proportion of the assembly (%)
**Class I**		
**LTR retrotransposons**		
Gypsy	448,883,024	33.58
Copia	156,347,966	11.69
ERV	1,312,713	0.10
Caulimovirus	1,463,548	0.11
Unclasssified	756,480	0.06
**LINE**		
R2	1,206,051	0.09
RTE	1,692,267	0.13
Jockey	742,469	0.06
L1	21,604,281	1.62
CRE	3,298,455	0.25
Unclasssified	1,234,992	0.09
**SINE**	15,186	0.00
**Class II**		
CMC	48,617,566	3.64
hAT	15,146,731	1.13
MuDR	12,738,187	0.95
TcMar	8,147,037	0.61
En	7,900,599	0.59
PIF	3,363,523	0.25
Harbinger	1,468,810	0.11
Maverick	1,266,066	0.09
Unclasssified	2,019,151	0.15
**Total**	73,940,212	55.30
