# Supplementary material for: A high-quality genome assembly of quinoa provides insights into the molecular basis of salt bladder-based salinity tolerance and the exceptional nutritional value
Source: Cell Res. 2017 Oct 10;27(11):1327–40. doi: 10.1038/cr.2017.124 (PMC5674158; doi:10.1038/cr.2017.124)
Supplement: Supplementary information, Table S6 — Summary of SSRs (simple sequence repeats) identified in quinoa [file cr2017124x22.pdf]

Table S6. Summary of SSRs (simple sequence repeats) identified in quinoa

Motif	Counts	Average length (bp)	Average mismatches (bp)	Counts/Mbp
Mononucleotide	121,810	22.8	0.4	91.1
Dinucleotide	44,872	42.3	1	33.6
Trinucleotide	76,221	39.5	1.4	57
Tetranucleotide	46,847	20.2	0.3	35.1
Pentanucleotide	77,059	20.6	0.4	57.7
Hexanucleotide	25,955	26.6	0.7	19.4
**Total**	**392,764**	**28.7**	**0.7**	**49**
