# Supplementary material for: A high-quality genome assembly of quinoa provides insights into the molecular basis of salt bladder-based salinity tolerance and the exceptional nutritional value
Source: Cell Res. 2017 Oct 10;27(11):1327–40. doi: 10.1038/cr.2017.124 (PMC5674158; doi:10.1038/cr.2017.124)
Supplement: Supplementary information, Table S7 — A list of top SSRs identified in quinoa [file cr2017124x23.pdf]

Table S7. A list of top SSRs identified in quinoa

Motif	Counts	Average length	Average mismatches	Counts per Mb
A	119,416	22.87	0.37	89.35
AAT	42,557	45.84	1.88	31.84
AT	27,048	53.00	1.33	20.24
AAAT	23,566	20.09	0.32	17.63
AAAAT	18,284	20.27	0.37	13.68
AACTG	14,320	28.06	1.00	10.71
AG	11,520	26.60	0.46	8.62
AATT	9,619	20.65	0.24	7.20
AAC	8,170	30.20	0.92	6.11
AAG	7,513	46.89	0.83	5.62
AAAAG	7,168	18.32	0.23	5.36
AAATT	6,609	18.12	0.11	4.94
AC	6,280	25.27	0.52	4.70
ATC	5,542	21.75	0.52	4.15
ACC	5,067	20.62	0.54	3.79
AAAAAT	4,298	23.58	0.45	3.22
AAAG	3,216	18.88	0.28	2.41
AGCCT	2,552	15.15	0.00	1.91
AGG	2,547	22.14	0.71	1.91
AGC	2,525	20.67	0.47	1.89
C	2,394	20.45	0.21	1.79
AATC	2,069	18.29	0.20	1.55
ACT	1,880	61.79	1.92	1.41
AATAAC	1,836	36.24	1.86	1.37
AAACC	1,810	17.35	0.12	1.35
AAAAC	1,760	19.16	0.27	1.32
ACGGC	1,660	16.48	0.03	1.24
AAAC	1,601	18.57	0.25	1.20
AAACT	1,568	20.86	0.32	1.17
AGAGC	1,370	21.20	0.51	1.03
AAAAAG	1,332	23.24	0.45	1.00
ATCAG	1,172	20.07	0.29	0.88
AAGAG	1,093	21.65	0.84	0.82
ATAC	1,050	28.92	0.63	0.79
AGAGG	1,034	19.67	0.27	0.77
AATCAT	1,018	25.20	0.58	0.76
ACCCC	1,003	19.17	0.22	0.75
Others	39,238			
