# Supplementary material for: A high-quality genome assembly of quinoa provides insights into the molecular basis of salt bladder-based salinity tolerance and the exceptional nutritional value
Source: Cell Res. 2017 Oct 10;27(11):1327–40. doi: 10.1038/cr.2017.124 (PMC5674158; doi:10.1038/cr.2017.124)
Supplement: Supplementary information, Table S8 — Statistics of predicted quinoa gene models at each step of the annotation pipeline [file cr2017124x24.pdf]

Table S8. Statistics of predicted quinoa gene models at each step of the annotation pipeline

Method	Gene number	Average Length				Exon per Gene
		Gene	CDS	Exon	Intron	
Homology						
A. hypochondriacus	75,952	2595.4	914.2	267.4	695.1	3.4
A. thaliana	68,880	2320.5	892.7	274.8	635.2	3.2
T. salsuginea	73,610	2191.7	842.4	272.9	646.7	3.1
S. oleracea	135,497	1944.7	817.0	288.9	616.8	2.8
B. vulgaris	160,859	1869.5	791.3	303.8	671.9	2.6
ab initio						
Fgenesh	48,208	3955.5	1197.5	203.8	565.8	5.9
Augustus	63,690	2622.8	1045.8	218.9	417.5	4.8
Genescan	54,919	13792.9	1219.3	205.7	2551.4	5.9
GlimmerHMM	66,627	2280.4	879.1	226.7	487.1	3.9
SNAP	101,308	1296.2	636.7	203.6	310.0	3.1
mRNA-seq	51,172	3408.1	1104.1	231.2	534.1	4.6
GLEAN	54,438	3547.6	1096.3	232.0	560.7	4.8
