# Supplementary material for: A high-quality genome assembly of quinoa provides insights into the molecular basis of salt bladder-based salinity tolerance and the exceptional nutritional value
Source: Cell Res. 2017 Oct 10;27(11):1327–40. doi: 10.1038/cr.2017.124 (PMC5674158; doi:10.1038/cr.2017.124)
Supplement: Supplementary information, Table S9 — Statistics of noncoding RNAs (ncRNA) identified in the quinoa genome [file cr2017124x25.pdf]

Table S9. Statistics of noncoding RNAs (ncRNA) identified in the quinoa genome

Type	Copy Number	Average length (bp)	Total length (bp)	Percent genome
miRNA	192	192	24,160	<0.01
tRNA	2934	2934	215,855	0.02
rRNA				
18S	75	75	40,787	<0.01
28S	139	139	16,186	<0.01
5.8S	25	25	3,720	<0.01
5S	1071	1071	108,263	0.01
snRNA	5922			
CD-box	5565	5565	588,244	0.04
HACA-box	85	85	11,254	<0.01
splicing	272	272	39,465	<0.01
**Total**	**10,358**		**1,047,934**	**0.09**
