# Supplementary material for: A high-quality genome assembly of quinoa provides insights into the molecular basis of salt bladder-based salinity tolerance and the exceptional nutritional value
Source: Cell Res. 2017 Oct 10;27(11):1327–40. doi: 10.1038/cr.2017.124 (PMC5674158; doi:10.1038/cr.2017.124)
Supplement: Supplementary information, Table S10 — Summary of quinoa genes with assigned (predicted) functions [file cr2017124x26.pdf]

Table S10. Summary of quinoa genes with assigned (predicted) functions

	**Number**	**Percent(%)**
Total predicted genes	54,459	100.0
Annotated		
InterPro	41,060	75.4
GO	46,264	85.0
KEGG	27,486	50.4
Swissprot	35,708	65.6
TrEMBL	47,862	87.9
**Total**	**52,058**	**95.6**
