# Supplementary material for: A high-quality genome assembly of quinoa provides insights into the molecular basis of salt bladder-based salinity tolerance and the exceptional nutritional value
Source: Cell Res. 2017 Oct 10;27(11):1327–40. doi: 10.1038/cr.2017.124 (PMC5674158; doi:10.1038/cr.2017.124)
Supplement: Supplementary information, Table S11 — Summary of Cq genes identified in BUSCO v2 embryophyta gene set [file cr2017124x27.pdf]

Table S11. Summary of Cq genes identified in BUSCO v2 embryophyta gene set

Type	This study		(Jarvis et al. 2017)	
	**Number**	**Percent**	**Number**	**Percent**
Complete BUSCOs (C)	1344	93.3	1318	91.6
Complete and single-copy BUSCOs (S)	392	27.2	361	25.1
Complete and duplicated BUSCOs (D)	952	66.1	957	66.5
Fragmented BUSCOs (F)	23	1.6	25	1.7
Missing BUSCOs (M)	73	5.1	97	6.7
Total BUSCO groups* searched	1440	---	1440	---
