# Supplementary material for: A high-quality genome assembly of quinoa provides insights into the molecular basis of salt bladder-based salinity tolerance and the exceptional nutritional value
Source: Cell Res. 2017 Oct 10;27(11):1327–40. doi: 10.1038/cr.2017.124 (PMC5674158; doi:10.1038/cr.2017.124)
Supplement: Supplementary information, Table S12 — Estimated accuracy of gene prediction [file cr2017124x28.pdf]

Table S12. Estimated accuracy of gene prediction

	High-expression transcripts		mRNA (NCBI)	
	**Sensitivity**	**Specificity**	**Sensitivity**	**Specificity**
Gene	69.58%	78.39%	21.67%	31.43%
Transcript	42.25%	75.74%	21.67%	31.43%
Exon	71.92%	92.62%	69.74%	43.09%
Nucleotide	95.27%	98.44%	94.18%	55.27%
