# Supplementary material for: A high-quality genome assembly of quinoa provides insights into the molecular basis of salt bladder-based salinity tolerance and the exceptional nutritional value
Source: Cell Res. 2017 Oct 10;27(11):1327–40. doi: 10.1038/cr.2017.124 (PMC5674158; doi:10.1038/cr.2017.124)
Supplement: Supplementary information, Table S13 — Comparison of Cq_real_v1.0 with two published quinoa assemblies [file cr2017124x29.pdf]

Table S13. Comparison of Cq_real_v1.0 with two published quinoa assemblies

	Cq_real_v1.0 (This study)		Cqu_r1.0 (Yasui et al. 2016)		ASM168347v1 (Javis et al. 2017)	
	**Number**	**Size**	**Number**	**Size**	**Number**	**Size**
**Assembly features**						
Total Scaffolds (≥100bp)	3,184	1,337 Mb	24,845	1,087 Mb	3,486	1,385 Mb
Scaffold N50	373	1,162 Kb		86 Kb	105	3,847 Kb
Scaffold N90	1,087	423 Kb			439	250 Kb
Longest Scaffold		5,398 Kb		641 Kb		23,816 Kb
Average Scaffold length		420 Kb		43 Kb		397 kb
GC content	37.00%		36.90%		36.90%	
Undetermined bases	0.87%	11,704 Kb	2.61%	28,385 Kb	4.56%	63,176 Kb
**Genome annotation**						
Total repetitive sequences	64.50%	863 Mb	61.50%	668 Mb	64.02%	879 Mb
Gene models	54,438	61.9 Mbp*	226,647	190.5 Mbp*	44,776	57.1 Mbp

* The total length of coding sequences
