# Supplementary material for: A high-quality genome assembly of quinoa provides insights into the molecular basis of salt bladder-based salinity tolerance and the exceptional nutritional value
Source: Cell Res. 2017 Oct 10;27(11):1327–40. doi: 10.1038/cr.2017.124 (PMC5674158; doi:10.1038/cr.2017.124)
Supplement: Supplementary information, Table S14 — Enriched GO terms in quinoa-specific orthologous groups [file cr2017124x30.pdf]

Table S14. Enriched GO terms in quinoa-specific orthologous groups

GO ID	p Value	q Value	Term	Classification
GO:0010329	5.30E-10	2.80E-07	auxin efflux transmembrane transporter activity	MF
GO:0005515	8.86E-07	1.41E-04	protein binding	MF
GO:0003677	1.54E-06	2.22E-04	DNA binding	MF
GO:0003723	8.04E-06	9.12E-04	RNA binding	MF
GO:0017016	2.26E-05	1.91E-03	Ras GTPase binding	MF
GO:0016709	5.55E-05	3.54E-03	oxidoreductase activity, acting on paired donors, with incorporation or reduction o	MF
GO:0046872	6.24E-05	3.54E-03	metal ion binding	MF
GO:0004252	6.35E-05	3.54E-03	serine-type endopeptidase activity	MF
GO:0042626	6.97E-05	3.69E-03	ATPase activity, coupled to transmembrane movement of substances	MF
GO:0016740	1.28E-04	6.54E-03	transferase activity	MF
GO:0047134	1.77E-04	7.40E-03	protein-disulfide reductase activity	MF
GO:0001727	2.63E-04	9.72E-03	lipid kinase activity	MF
GO:0009840	5.59E-11	4.43E-08	chloroplastic endopeptidase Clp complex	CC
GO:0005739	4.28E-09	1.36E-06	mitochondrion	CC
GO:0009507	9.61E-08	2.18E-05	chloroplast	CC
GO:0009532	1.38E-07	2.73E-05	plastid stroma	CC
GO:0005634	4.74E-07	8.36E-05	nucleus	CC
GO:0005829	2.12E-06	2.81E-04	cytosol	CC
GO:0005737	6.04E-06	7.38E-04	cytoplasm	CC
GO:0005622	1.82E-05	1.81E-03	intracellular	CC
GO:0032045	1.77E-04	7.40E-03	guanyl-nucleotide exchange factor complex	CC
GO:0044599	2.63E-04	9.72E-03	AP-5 adaptor complex	CC
GO:0002238	2.64E-09	1.05E-06	response to molecule of fungal origin	BP
GO:0006351	1.01E-05	1.07E-03	transcription, DNA-templated	BP
GO:0044550	2.31E-05	1.91E-03	secondary metabolite biosynthetic process	BP
GO:0006355	2.40E-05	1.91E-03	regulation of transcription, DNA-templated	BP
GO:0090602	2.41E-05	1.91E-03	sieve element enucleation	BP
GO:0006624	2.52E-05	1.91E-03	vacuolar protein processing	BP
GO:0090603	3.24E-05	2.34E-03	sieve element differentiation	BP
GO:0035957	6.47E-05	3.54E-03	positive regulation of starch catabolic process by positive regulation of transcriptio	BP
GO:1900524	6.47E-05	3.54E-03	positive regulation of flocculation via cell wall protein-carbohydrate interaction by	BP
GO:1900461	6.47E-05	3.54E-03	positive regulation of pseudohyphal growth by positive regulation of transcription	BP
GO:0036095	6.47E-05	3.54E-03	positive regulation of invasive growth in response to glucose limitation by positive	BP
GO:0048235	1.35E-04	6.68E-03	pollen sperm cell differentiation	BP
GO:0006810	1.61E-04	7.40E-03	transport	BP
GO:0051966	1.77E-04	7.40E-03	regulation of synaptic transmission, glutamatergic	BP
GO:0060079	1.77E-04	7.40E-03	excitatory postsynaptic potential	BP
GO:1900452	1.77E-04	7.40E-03	regulation of long term synaptic depression	BP
GO:0010540	1.96E-04	7.97E-03	basipetal auxin transport	BP
GO:0010981	2.45E-04	9.72E-03	regulation of cell wall macromolecule metabolic process	BP
GO:0045892	2.63E-04	9.72E-03	negative regulation of transcription, DNA-templated	BP
