# Supplementary material for: A high-quality genome assembly of quinoa provides insights into the molecular basis of salt bladder-based salinity tolerance and the exceptional nutritional value
Source: Cell Res. 2017 Oct 10;27(11):1327–40. doi: 10.1038/cr.2017.124 (PMC5674158; doi:10.1038/cr.2017.124)
Supplement: Supplementary information, Table S15 — List of other genomes used in this study [file cr2017124x31.pdf]

Table S15. List of other genomes used in this study

Species	Version	Source
A. hypochondriacus	Phytozome 11	JGI (https://phytozome.jgi.doe.gov)
A. thaliana	Phytozome 11	JGI (https://phytozome.jgi.doe.gov)
F. esculentum	Version 1.0	BGDB (http://buckwheat.kazusa.or.jp)
T. salsuginea	Phytozome 11	JGI (https://phytozome.jgi.doe.gov)
S. oleracea	ASM200726v1	GeneBank (https://www.ncbi.nlm.nih.gov)
G. max	Phytozome 11	JGI (https://phytozome.jgi.doe.gov)
Z. may	Phytozome 11	JGI (https://phytozome.jgi.doe.gov)
O. sativa	Phytozome 11	JGI (https://phytozome.jgi.doe.gov)
Aegilops tauschii	Aet_MR_1.0	GeneBank (https://www.ncbi.nlm.nih.gov)
B. vulgaris	RefBeet-1.2.2	GeneBank (https://www.ncbi.nlm.nih.gov)
