# Supplementary material for: A high-quality genome assembly of quinoa provides insights into the molecular basis of salt bladder-based salinity tolerance and the exceptional nutritional value
Source: Cell Res. 2017 Oct 10;27(11):1327–40. doi: 10.1038/cr.2017.124 (PMC5674158; doi:10.1038/cr.2017.124)
Supplement: Supplementary information, Data S1 — Supplemental methods [file cr2017124x37.pdf]

Supplementary information, Data S1 Supplemental methods

Genome assembly

Three intermediate assembly versions for the quinoa genome were generated using Illumina reads (v0.1) and PacBio reads (v0.2 and v0.3) separately. Then the three assemblies were merged together using the HABOT2 software (1gene Corp., Hangzhou, China; https://github.com/asarum/HABOT2) and a final round of scaffolding and gap filling was performed using Illumina reads to obtain Cq assembly v1.0. Detailed protocols are described below.

Filtered Illumina reads from two PCR-free libraries, with average insert sizes of ~380 bp and ~450 bp respectively, were used to construct contigs using the DISCOVAR *de novo* software [1, 2]. Reads from the two medium-size mate pair libraries (Supplementary Table 1) were then used for scaffolding with SSPACE 3.0 [3]. Gaps inside the constructed scaffolds were filled using GapCloser v1.12 module from SOAPdenovo2 [4]. The resulting assembly v0.1 has a scaffold N50 of 49.5kb and contig N50 of 26.1kb (Supplementary Table 3). Parameters used for each software are listed below:

```
Discovar:
DiscovarDeNovo READS=./data/*fastq.gz  OUT_DIR=Assembly

SSPACE:
perl SSPACE_Standard_v3.0.pl -l lib.cfg -s a.lines.fasta  -T 8

GapCloser:
GapCloser -o closegap.fa -b lib.cfg -a closeGap.fa  -t 16
```

Falcon v0.3 was used to assemble Cq assembly v0.2. The config file for running Falcon v0.3:

```
Cq.cfg:

input_fofn = input.fofn
input_type = raw
```

```
length_cutoff = 8000
```

```
length_cutoff_pr = 5000
pa_HPCdaligner_option =  -v -dal128 -M32 -e.70 -l1000 -s1000
pa_DBsplit_option = -x500 -s400
falcon_sense_option = --output_multi --min_idt 0.70 --min_cov 4 --
local_match_count_threshold 2 --max_n_read 200 --n_core 10
overlap_filtering_setting = --max_diff 500 --max_cov 500 --min_cov 2
--bestn 10 --n_core 6
```

We then used Canu to obtain Cq assembly v0.3 that has a contig N50 of ~51 kb and an assembly size of ~1.43Gb (Supplementary Table 3).

```
canu -p Cq_assem -d Cq_assem_34X genomeSize=1480m useGrid=remote -
pacbio-raw ./Data/Cq.Pcabio.fa
```

Then HABOT2 was used to combine contigs over 1 kb in length from the above three assemblies and to construct a new contig set. HABOT2 contains 4 modules:

a. Graph module. This module counts k-mer frequencies and extracts the **unique k-mers** from Illumina reads. A unique k-mer is theoretically defined as a k-bp sequence that occurs just once in a haploid genome and is calculated following a Poisson model as described previously [5]. Using unique k-mers, instead of all the k-mers, for graph construction minimizes the effects of error-prone repeats and increases computation speed.
b. Alignment module. This module is use for an all-to-all alignment between PacBio contigs and Illumina contigs. By using unique k-mers it performs the alignment much faster than blasr [6] and is of high accuracy.
c. Duplication remove module. When two sequences have common uinque k-mers that exceed a cutoff (default is 0.5), the shorter sequence is removed.
d. *De novo* module. This module `calls` the above 3 modules and performs hybrid assembly.

For the Cq assembly, we extracted unique 17-mers from the PCR-free Illumina libraries. Overlaps among contigs from different intermediate assembly versions were

identified using the alignment module. Then the OLC (Overlap Layout Consensus) graph was built for the overlapped contigs. The connection from contig A to contig B is dropped in case of the following situation:

```
(1) Contig A's best connection is contig B
(2) Contig B's best connection is contig C
(3) A does not overlap with C
```

After the merge of overlapped contigs, duplicated regions in the contig set were removed. Finally scaffolding and gap closure were performed on the new contig set using Illumina mate pair reads with SSPACE v3.0 and GapCloser v1.12 (both with default parameters).

Next we utilized the PE250 reads from two PCR-free libraries to correct for assembly errors. Filtered reads were aligned to the assembly using bwa mem with default parameters. Next the GATK package [7] was used to identify variants. The order of commands and corresponding parameters used were as following: IndelRealigner; UnifiedGenotyper --read_filter BadCigar -glm BOTH -stand_call_conf 30.0 -stand_emit_conf 0; VariantFiltration --filterExpression "QD<20.0 || ReadPosRankSum <-8.0 || FS>10.0 || QUAL<30 --filterName LowQualFilter; BaseRecalibrator; PrintReads; UnifiedGenotyper --read_filter BadCigar -glm BOTH -stand_call_conf 30.0 -stand_emit_conf 0; VariantFiltration --filterExpression "QD < 20.0 || ReadPosRankSum < -8.0 || FS > 10.0 || QUAL < 30 --filterName LowQualFilter. Then an in-house perl script was used to correct for the highly-confident errors identified. This process was reiterated for a total of 5 times to generate assembly v1.0.

Specific HABOT2 commands are listed below:

a) Extract unique 17-mers from Illumina reads:

```
perl Graph.pl pipe -i fq.lst -m 2 -k 17 -s 1,3 -d Kmer_17
```

b) Config file for hybrid assembly using the de novo module:

```
# the input file list, in fasta format
[pb_lst] file.lst

# Data type:
# 1: the uncorrected data
# 2: the corrected data
[pb_type] 2

# filter read length
[filt_len] 1000

# genome size in Magebase
[genome_size] 1500

# unique kmer, kmer size
[unique_kmer] kmer_17.bit 17

# Align parameters
# Col.1, kmer size
# Col.2: the scope for find anchor, -1 is for all length
# Col.3: align mode
#         1: for uncorrected reads
#         2: for corrected reads
# Col.4: filter score below this value
[strategy] 17 -1    2   0.8
[strategy] 17 -1    1   0.9
[strategy] 35 1000  1   0.9
[strategy] 35 -1    1   0.9

# project name
[pro_name] CQ

# qsub parameters
[queue] dna.q,rna.q,reseq.q
[Project] og
[max_job] 50
[thread] 8
```

Then run the command:

```
perl Denovo.pl input.cfg &
```

Assessment of the assembly

Alignment of PE250 reads (from the two PCR-free libraries) to Cq_real_v1.0 were performed using bwa mem with default parameters. Then samtools and bcftools were used for SNP calling and summarization.

A 40-kb fosmid library for quinoa was constructed using CopyControl Fosmid Library Production Kit (Epicenter). Then 10 single colonies were picked and cultured in 100 mL LB medium. The 10 fosmids were then extracted using a Plasmid Midi Kit (Qiagen), mixed in equal quantity and used for a 20-kb PacBio library preparation. Library preparation and sequencing were performed at Tianjin Biochip Corporation. Falcon v0.3.0 was used for the *de novo* assembly of fosmid sequences with default parameters. After removing plasmid sequences, the contigs were then aligned to Cq_real_v1.0 using blastn with parameters –task blastn-short. The results were summarized manually.

Chloroplast Genome Annotation

Annotation of the chloroplast genome was performed separately using DOGMA [8] and CpGAVAS [9] with the following parameters: blast E-value cutoff - 1e-10, maximum target hit number – 10, and maximum length of tRNA intron and variable region - 116 bp. Then outputs from both software were integrated by retaining the longer opening read frame (ORF) with an in-house Perl script. The predicted start/stop codons and the exon-intron boundaries for intron-containing genes were manually examined and curated. The map of the chloroplast genome was generated using GenomeVx [10] followed by some manual adjustment.

Annotation of Repeats

Both homology-based and *de novo* approaches were used for repeat annotation. Three complementary software programs, LTR_FINDER [11], PILER [12], RepeatModeler

[13], were used with default parameters to generate a *de novo* repeat library for quinoa. These programs use complementary methods to predict repeats. LTR_FINDER retrieves full-length LTR retrotransposons; PILER searches for repeats in the genome by aligning the genome sequence to itself; RepeatModeler uses two complementary repeat prediction programs, RECON and RepeatScout, to identify repeat element boundaries and family relationships. This *de novo* repeat library was then used together with Repbase [14] for homology search of repeats using RepeatMasker [15].

Gene Prediction and Annotation

Three independent approaches, including homology search, ab initio prediction and reference guided transcriptome assembly were used for gene prediction in a repeat-masked genome. Evidence from the three approaches were then merged using GLEAN to generate the final gene set.

Homology-based gene prediction. Putative open reading frames (ORFs) in the quinoa genome were identified by aligning the protein sequences of *A. thaliana*, *Thellungiella salsuginea*, *Beta vulgaris*, *Spinacia oleracea*, *Amaranthus hypochondriacus*, and *Fagopyrum esculentum* (Supplementary Table 15) to Cq_real_v1.0 using TBLASTN with an E-value cutoff of 1e−5. We next extracted those ORF regions containing introns from the genome, including 2,000-bp extensions at both ends, and again aligned protein sequences from other species to these DNA fragments using GeneWise [16] with parameters: –trev –sum –genesf.

Ab initio gene prediction. AUGUSTUS v2.5.5 [17] and SNAP (version 2006-07-28) [18] with default parameters were utilized for *de novo* gene prediction with gene model parameters trained with *A. thaliana* TAIR10 genome. Short coding sequences that were less than 150 bp in length were discarded.

Transcriptome-assisted gene prediction. We used TopHat [19] to map filtered mRNA-seq reads to Cq_real_v1.0 to identify exonic regions and intron-exon boundaries with the following parameters: -p 4 -max-intron-length 20,000 -m 1 -r 20 -mate-std-dev 20. Cufflinks [19] was then used to assemble the alignments into transcripts with the parameters: –I 20,000 –p 4.

Results derived from the above three approaches were integrated to generate a consensus gene set using GLEAN with default parameters [20]. The probabilistic confidence score generated by GLEAN was used to reflect the consistency among different sources of evidence.

Assessment of gene models

The completeness of gene prediction was assessed by comparing the quinoa protein sequences to the 1,411 embryophyta single copy orthologs in BUSCO v2 (Benchmarking Universal Single-Copy Orthologs) [21] with a BLAST E-value cutoff of 1e-5.

The predicted gene models were further assessed using Eval v2.2.8 [22]. Two sets of “gold standard” genes were used: the 56 mRNA sequences of *C. quinoa* retrieved from the NCBI nucleotide database and high-expression transcripts based on the mRNA-seq data. For high-expression transcripts, Trinity was used for *de novo* transcript assembly in the genome guided mode [23] with combined mRNA-seq data from 8 types of quinoa tissue and the transcripts with a sequencing coverage higher than 100 were retained. Maker v2.31.9 was used to map the assembled transcripts to Cq_real_v1.0 to generate the gene model in gff format.

Function annotation of genes

To assign gene functions, the predicted quinoa protein sequences were searched against five protein/function databases: InterPro, GO, KEGG, Swiss-Prot and

TrEMBL. The Interpro database search was performed using InterproScan with parameters: -f TSV –dp –gotermes -iprlookup –pa. For the 4 databases, BLAST searches using the quinoa protein sequences as query were performed with an E-value cutoff of 1e-05. Results from the 5 database searches were then concatenated.

For GO term enrichment analysis, Fisher's exact test was performed and the p-value was adjusted for multiple testing using the BH method.

References

1. Weisenfeld NI, Yin S, Sharpe T, et al. Comprehensive variation discovery in single human genomes. *Nat Genet* 2014; **46:**1350-1355.
2. Love RR, Weisenfeld NI, Jaffe DB, Besansky NJ, Neafsey DE. Evaluation of DISCOVAR de novo using a mosquito sample for cost-effective short-read genome assembly. *BMC Genomics* 2016; **17:**187.
3. Boetzer M, Henkel CV, Jansen HJ, Butler D, Pirovano W. Scaffolding pre-assembled contigs using SSPACE. *Bioinformatics* 2011; **27:**578-579.
4. Luo R, Liu B, Xie Y, et al. SOAPdenovo2: an empirically improved memory-efficient short-read de novo assembler. *Gigascience* 2012; **1:**18.
5. You M, Yue Z, He W, et al. A heterozygous moth genome provides insights into herbivory and detoxification. *Nat Genet* 2013; **45:**220-225.
6. Chaisson MJ, Tesler G. Mapping single molecule sequencing reads using basic local alignment with successive refinement (BLASR): application and theory. *BMC Bioinformatics* 2012; **13:**238.
7. Van der Auwera GA, Carneiro MO, Hartl C, et al. From FastQ data to high confidence variant calls: the Genome Analysis Toolkit best practices pipeline. *Curr Protoc Bioinformatics* 2013; **43:**11 10 11-33.
8. Wyman SK, Jansen RK, Boore JL. Automatic annotation of organellar genomes with DOGMA. *Bioinformatics* 2004; **20:**3252-3255.
9. Liu C, Shi L, Zhu Y, et al. CpGAVAS, an integrated web server for the annotation, visualization, analysis, and GenBank submission of completely sequenced chloroplast genome sequences. *BMC Genomics* 2012; **13:**715.
10. Conant GC, Wolfe KH. GenomeVx: simple web-based creation of editable circular chromosome maps. *Bioinformatics* 2008; **24:**861-862.
11. Xu Z, Wang H. LTR_FINDER: an efficient tool for the prediction of full-length LTR retrotransposons. *Nucleic Acids Res* 2007; **35:**W265-268.
12. Edgar RC, Myers EW. PILER: identification and classification of genomic repeats. *Bioinformatics* 2005; **21 Suppl 1:**i152-158.
13. Price AL, Jones NC, Pevzner PA. De novo identification of repeat families in large genomes. *Bioinformatics* 2005; **21 Suppl 1:**i351-358.
14. Bao W, Kojima KK, Kohany O. Repbase Update, a database of repetitive elements in eukaryotic genomes. *Mob DNA* 2015; **6:**11.
15. Tarailo-Graovac M, Chen N. Using RepeatMasker to identify repetitive elements in genomic sequences. *Curr Protoc Bioinformatics* 2009; **Chapter 4:**Unit 4 10.

16. Birney E, Clamp M, Durbin R. GeneWise and Genomewise. *Genome Res* 2004; **14:**988-995.
17. Stanke M, Keller O, Gunduz I, Hayes A, Waack S, Morgenstern B. AUGUSTUS: ab initio prediction of alternative transcripts. *Nucleic Acids Res* 2006; **34:**W435-439.
18. Korf I. Gene finding in novel genomes. *BMC Bioinformatics* 2004; **5:**59.
19. Trapnell C, Williams BA, Pertea G, et al. Transcript assembly and quantification by RNA-Seq reveals unannotated transcripts and isoform switching during cell differentiation. *Nat Biotechnol* 2010; **28:**511-515.
20. Elsik CG, Mackey AJ, Reese JT, Milshina NV, Roos DS, Weinstock GM. Creating a honey bee consensus gene set. *Genome Biol* 2007; **8:**R13.
21. Simao FA, Waterhouse RM, Ioannidis P, Kriventseva EV, Zdobnov EM. BUSCO: assessing genome assembly and annotation completeness with single-copy orthologs. *Bioinformatics* 2015; **31:**3210-3212.
22. Keibler E, Brent MR. Eval: a software package for analysis of genome annotations. *BMC Bioinformatics* 2003; **4:**50.
23. Grabherr MG, Haas BJ, Yassour M, et al. Full-length transcriptome assembly from RNA-Seq data without a reference genome. *Nat Biotechnol* 2011; **29:**644-652.
